# Cognitive Control Processes and Defense Mechanisms That Influence Aggressive Reactions: Toward an Integration of Socio-Cognitive and Psychodynamic Models of Aggression

**DOI:** 10.3389/fnhum.2021.751336

**Published:** 2022-01-14

**Authors:** Jean Gagnon, Joyce Emma Quansah, Paul McNicoll

**Affiliations:** ^1^Department of Psychology, University of Montreal, Montréal, QC, Canada; ^2^Centre for Interdisciplinary Research in Rehabilitation of Greater Montreal (CRIR), Montreal, QC, Canada; ^3^Laboratoire d'électrophysiologie en neuroscience sociale (LENS), Université de Montréal, Montréal, QC, Canada

**Keywords:** aggression, hostile attribution bias, control process, response inhibition control, defense mechanisms, psychological regulation, event-related potentials (ERP)

## Abstract

Research on cognitive processes has primarily focused on cognitive control and inhibitory processes to the detriment of other psychological processes, such as defense mechanisms (DMs), which can be used to modify aggressive impulses as well as self/other images during interpersonal conflicts. First, we conducted an in-depth theoretical analysis of three socio-cognitive models and three psychodynamic models and compared main propositions regarding the source of aggression and processes that influence its enactment. Second, 32 participants completed the Hostile Expectancy Violation Paradigm (HEVP) in which scenarios describe a hostile vs. non-hostile social context followed by a character's ambiguous aversive behavior. The N400 effect to critical words that violate expected hostile vs. non-hostile intent of the behavior was analyzed. Prepotent response inhibition was measured using a Stop Signal task (SST) and DMs were assessed with the Defense Style Questionnaire (DSQ-60). Results showed that reactive aggression and HIA were not significantly correlated with response inhibition but were significantly positively and negatively correlated with image distorting defense style and adaptive defense style, respectively. The present article has highlighted the importance of integrating socio-cognitive and psychodynamic models to account for the full complexity underlying psychological processes that influence reactive aggressive behavior.

## Introduction

Severe negative consequences can result from aggressive behaviors and motivate researchers to study the factors that may prevent them. Among these factors, components of cognitive control processes are the primary candidates in socio-cognitive models of reactive aggression. For example, following a social provocation, *inhibitory control* processes are thought to suppress hostile thoughts before a person behaves aggressively (Wilkowski and Robinson, 2008). However, if the purpose of exerting this effort is not to suppress hostile thoughts but rather to transform them so that they become less threatening to the person themselves, then the psychological processes underlying the aggressive response may involve different mechanisms (e.g., defense mechanisms). For example, what if the person perceives their thoughts as coming from an outside source, from another person who has not provoked them (psychodynamic process called *projection*), how will they respond? What happens if the anger toward the person transforms into an opposite feeling such as kindness (*reaction-formation*), or into abstract thoughts about anger in human relations more generally (*intellectualization*)? Or, what if the perception of the other person rapidly oscillates from an extremely negative image to an extremely positive image (*splitting*)? These questions demonstrate the complexity of processes involved in changing hostile thoughts, emotions, and behaviors, and have all been addressed in psychodynamic models of personality. Here, defense mechanisms (DMs) represent the psychological processes that mediate the person's reaction and have been the subject of empirical studies over the last 50 years. While the role they play in modifying certain human behaviors has received little attention from the socio-cognitive literature in the past, there has recently been a growing interest in developing integrative models that connect psychodynamic data and cognitive neuroscience to enrich our understanding of the mind and brain (Axmacher et al., 2014; di Giannantonio et al., 2020).

Inspired by this scientific approach, the goal of the present study is to integrate cognitive control processes as conceptualized in socio-cognitive models of aggression with the construct of DMs in psychodynamic models of personality. The implications of this work could promote a better understanding of some of the psychological processes that influence aggression. For these constructs to be comparable, the challenge is to identify the distinct cognitive processes that are compatible with each of the DMs. The present article is a first step toward this goal. In part 1 we review three socio-cognitive and three psychodynamic models of aggression and compare their theoretical explanations for processes that influence it. In part 2, we conducted an empirical study to verify the relationships between reactive aggression and the hostile intent attribution (HIA) (i.e., a type of cognitive interpretive bias in socio-cognitive models), as well as two key processes in socio-cognitive and psychodynamic models that influence aggressive reactions (prepotent response inhibition and DMs).

## Part 1: Theoretical Analysis of Socio-Cognitive and Psychodynamic Models of Aggression

### Socio-Cognitive Models of Aggression

Socio-cognitive models of aggression developed from early social learning and social cognitive theories and expand on the idea that aggressive behavioral tendencies emerge from the development and activation of aggression-related scripts. More generally, scripts are the product of learning through observation and experiences in social environments and are stored in a person's long-term memory. Behavioral scripts contain information about how a person should behave in various social situations and are used to guide their interpretations of social stimuli and events, orientate their goals and decision making, shape their outcome expectations, mediate their behavior, and inform subsequent appraisals. From a socio-cognitive perspective, aggressive scripts develop from having learned that using aggressive strategies is an effective way of achieving our goals. Studies of aggression support the importance of distinguishing between different types of aggressive behaviors according to the goal that is pursued (Barratt et al., 1997). In reactive/impulsive aggression, which is characterized by uncontrolled or impulsive outbursts of anger, the primary desire is to hurt or injure the individual that represents a threat or a source of frustration (Berkowitz, 1993). In contrast, proactive/premeditated is relatively non-emotional, often premeditated or planned, and is motivated by the desire to achieve personal and/or social goals other than the target's harm (Dodge and Coie, 1987; Stanford et al., 2003). Over time, social situations that activate hostile thoughts and aggressive scripts provide ample opportunities for enacting and reinforcing these aggressive behaviors.

The following models build on this core paradigm and share a similar view of aggression as a social behavior that is primarily influenced by how a person thinks. As such, socio-cognitive models propose that inhibiting aggressive impulses requires a person to interrupt aggression-promoting hostile thoughts. Despite differences in terminology and general scope, each of the following models describes cognitive processes that may counteract the effects of automatic and erroneous attributional biases that enable aggression. We will begin with the model proposed by Crick and Dodge (1996) that describes how social information processes relate to aggressive behavior, followed by Anderson and Bushman's (2002) model addressing how these processes become automatic over time, and conclude with Wilkowski and Robinson's (2010) model that specifies how cognitive resources may counteract them and reduce aggressive impulses.

#### The Social Information Processing Model

Crick and Dodge originally developed the Social Information Processing model (SIP; Dodge, 1989; Crick and Dodge, 1996) as a framework for understanding social adjustment in children. They later reformulated the model and used it to explain why some individuals are prone to aggressive behavior. The SIP model proposes that individuals enter social situations and proceed by deploying a series of information processing steps. They begin by encoding situational and internal cues, then interpret them according to personalized scripts. After considering their goals and outcome expectations, they assess their ability to obtain them, select a response, and enact the behavior (Crick and Dodge, 1996). Given that response patterns are hypothesized to function according to previous learning of appropriate social behavior (i.e., cognitive scripts), the processes underlying “outcome expectations” represent the primary implementation of control processes. Crick and Dodge (1996) proposed that a child's repertoire of response strategies increases as they get older and learn adaptive ways to manage interpersonal conflicts. Therefore—assuming the person is motivated by positive social outcomes and feels confident about their ability to acquire them—thinking about this expected outcome will inhibit the selection of a (potentially) harmful or antisocial response.

Crick and Dodge (1996) proposed two possibilities for explaining why cognitive processes fail to inhibit an aggressive response. First, they proposed that some “aggressive children” have developed positive outcome expectations and feel confident about the use of aggression to resolve conflicts and achieve personal goals. They suggested the possibility that inhibitory processes have less of an effect for children who are less inclined to resolve interpersonal conflicts or consider social relationships as rewarding. The second possibility is that some children have a biased perception of social information and automatically interpret the intention of others as hostile. In this case, the aggression serves as a defensive reaction to a perceived threat, and inhibitory processes are (assumedly) never employed. Crick and Dodge's (1994, 1996) early model credited deficient SIP processes (encoding and interpreting) as the underlying reason a person habitually reacts aggressively and did not provide more explanatory detail (at least in this early model) about how automatic cognitive processes might be controlled. Fontaine and Dodge (2006) later developed a new model focusing on the response and decision step of the SIP (the RED model) to explain how individuals consider (or fail to consider) different response options during “on-line” judgments and decision-making before the behavioral enactment (Fontaine and Dodge, 2006). Also, they considered possible intervention techniques that involve teaching children to recognize internal cues (e.g., feelings of anger or hostility) and practice more self-control (Crick and Dodge, 1996).

#### The General Aggression Model

Anderson and Bushman (2002) developed a theoretical framework that integrated various other socio-cognitive models of aggression. The General Aggression Model (GAM; Anderson and Bushman, 2002) builds on the premise that habitual activation of aggression-related knowledge structures (i.e., scripts) shapes how an individual interprets and responds to others in social situations. The GAM model is divided into four main components: distal and developmental factors (biological, environmental, and personality variables), proximate internal state factors (cognition, affect, arousal), cognitive appraisal and decision-making processes, and thoughtful (i.e., controlled) or impulsive action. The model also includes a series of feedback processes that indicate how these four components influence each other and contribute to increase or decrease the development of an aggressive personality (Anderson and Carnagey, 2004).

A key component of the GAM model is the notion of aggressive priming. Briefly, the priming (in this case) is conceptualized as the activation of a thought, which then initiates a chain-like reaction involving the activation of associated thoughts, and results in making them more accessible (Collins and Loftus, 1975). Due to this process, aggressive thoughts that become repeatedly activated across multiple situations (or episodes) are thought to become easily accessible and contribute to the formation of HABs (Anderson and Bushman, 2002). According to this model, while activating aggression-related thoughts (e.g., hostile attribution) can increase feelings of anger and arousal, it may also take a more direct route and trigger an aggressive behavioral response. Like the SIP model, the GAM describes the initial encoding and interpretation processes as operating in an automatic fashion, without any conscious awareness or effort on the part of the person. Response selection processes are dependent on the value the person places on expected outcomes, as well as whether time and mental resources are sufficient for enacting the behavior. Control processes are *only* employed *if* these requirements are not met (e.g., if outcomes are too costly or insignificant, or when mentally fatigued), in which case the person engages in a process of reappraisal and considers alternative ways to interpret the situation. Importantly, reappraisals are controlled cognitive processes and require explicit, intentional effort. Like the SIP model, however, the GAM provides little information about how these cognitive processes operate to inhibit aggressive impulses.

#### The Integrative Cognitive Model

Wilkowski and Robinson (2010) proposed a model to explain how individual differences in distinct cognitive processes contribute to reactive aggression. The Integrative Cognitive Model (ICM) integrates socio-cognitive theories of personality and its approach to trait anger (Mischel and Shoda, 1995; Cervone and Shoda, 1999), proposing that individuals who have higher levels of trait anger are more emotionally reactive and prone to interpret ambiguous social situations as being hostile. Essentially, three cognitive processes underlie this assumption: hostile interpretation of situational inputs, rumination on hostile thoughts, and effortful control (controlled processes).

In developing the ICM, Wilkowski and Robinson were influenced by research suggesting that the degree to which the HAB increases reactive aggression is dependent on its ability to increase anger (Rudolph et al., 2004 in Wilkowski and Robinson, 2010). Accordingly, they proposed that when a person automatically attributes hostile intent, they become angrier. By selectively attending and reinforcing these hostile thoughts (i.e., rumination), their anger intensifies and increases the likelihood that they will respond aggressively. Apart from these automatic cognitive processes, the ICM also considers relevant controlled processes that operate to suppress or amplify them.

Wilkowski and Robinson incorporated the idea that individuals who are low in trait anger are more capable of recruiting the cognitive resources necessary to control hostile and angry thoughts (Wilkowski and Robinson, 2008). By “cooling down,” these individuals are thought to be better able to recruit limited-capacity cognitive resources and employ more controlled and effortful processes to counteract aggressive tendencies. In the ICM, effortful control is proposed to be the primary mechanism that self-regulates aggressive thoughts and behaviors. Wilkowski and Robinson wanted to offer a more dynamic view of effortful control, as previous conceptualizations described a trait-like cognitive resource that remains relatively dormant until utilized in specific situations involving cognitive conflicts (Botvinick et al., 2001). They were unclear on these effortful control processes; they did, however, describe three possible pathways through which they may operate. First, by interrupting activated hostile thoughts, they provide an opportunity for the person to reappraise situational inputs and may facilitate non-hostile interpretations. Second, by redirecting attentional processes away from the hostile thoughts, deleterious effects caused by rumination may be avoided. Lastly, they may directly suppress behavioral impulses and inhibit any visible indicators of anger (e.g., aggressive body language), which may also help decrease high levels of arousal.

Despite their limitations, all three of these models have been used to develop intervention strategies and evidence-based programs (Lochman and Wells, 2002; Hawkins and Cougle, 2013; Gilbert et al., 2017; Osgood et al., 2021). Each of these models acknowledge the relative contributions of situational and intrapersonal variables in driving these effects but varies in terms of specifying their role concerning cognitive control processes. Importantly, cognitive factors represent only part of the puzzle in our understanding of the underlying mechanisms that mediate harmful behaviors.

### Psychodynamic Models of Aggression

There are a plethora of psychodynamic models that have contributed to our understanding of one or several aspects of human aggression. They all share common features and, in a general sense, are models of personality in which we can find causal and motivational explanations for aggressive behavior. Importantly, psychodynamic models rest on the assumption that unconscious psychological processes are responsible for hostile propensities (also called aggressive impulses which encompass thoughts, feelings, or behaviors), as well as their counterparts, the DMs. Also, psychodynamic models of personality usually shed light on the motivations that underlie defensive operations and point to how they may be useful in countering aggressive impulses.

#### Freud's Views on Aggression

Until 1920, Sigmund Freud was reluctant to consider aggression a drive *per se* because he believed that the pressure that pushes a person into irrepressible action is characteristic of all drives (Laplanche and Pontalis, 2018). Rather, aggressive tendencies were conceptualized as feelings, attitudes, or behaviors. For Freud, ambivalence—the simultaneous presence of negative and positive feelings toward the same object—was present in all human relationships (Freud, 1898, 1905, 1912, 1915a, 1923). For example, we find the polarity of love (affection) and hate (aggressiveness) in the positive and negative transference from patient to therapist (i.e., in the repetition of unconscious infantile desires), which is expressed symbolically as rituals in obsessional neurosis (i.e., a destructive wish toward the person that is then magically canceled). We also see it during the developmental stages of the child's Oedipus complex, expressed as feelings of love and hostility toward a parent. In his first drive model (Freud, 1915a), love is derived from the sexual drive (libido) whereas hate is derived from the Ego drive (also called the self-preservative drive). Importantly, drives must be distinguished from biological instincts in that they are the amount of pressure or *work* somatic excitations put on mental functions (as opposed to bodily functions) to find a way to rid the pressure and elicit a discharge through a motor response. Because phenomena from the drive are internal and thus cannot be avoided with a flight response (as is the case with external dangers), mental action is required in response. Contrary to the Ego drives, which push the person to develop adaptive ways of interacting in their real-life environment and discover appropriate objects to reduce tension (e.g., food to satisfy hunger), the sexual drive can become a source of conflict for the Ego because its demands are unrealistic and satisfying its desires is either forbidden or inappropriate. Accordingly, *repression* is a DM that can be used to counteract the pressure of the sexual desires by pulling them back into the unconscious parts of the mind or blocking their access to the conscious mind (which would be unfathomable for the Ego drives, as satisfying their needs are necessary for the person's survival [Freud, 1915b]).

After being confronted with soldiers coming back from World War I who had traumatic nightmares, as well as seeing patients with other disorders who showed patterns of thoughts or behaviors that brought them suffering, Freud (1920) introduced a major revision to his drive model by proposing the existence of the death and life drives. Eros, the life drive, seeks to create and maintain vital units (forms of life) for the human species (through the sexual drives) and preserve the individual (self-preservative drives). In opposition, the death drive seeks the destruction of these units to return to an earlier state that existed before life, a state where all excitations (e.g., somatic tensions that manifest as unpleasant feelings) are reduced to zero. Because the death drive has no energy of its own, it borrows energy from the life drive to redirect the destruction outward, toward the external world. Consequently, Freud viewed the aggressive drive as resulting from a fusion between the life and death drives. In his second model of personality (Freud, 1923), both drives (life and death) belong to the Id, which now comprises sexual *and* aggressive desires that threaten the Ego. With the help of feelings of anxiety (a signal of potential danger; in the event of a forbidden desire is fulfilled and possibly result in reprimands from the Superego or punishment from an authority figure), the Ego then faces its conflict with the Id by making use of DMs. The drive coming from the Id (e.g., sexual, or aggressive desires in the unconscious mind) is composed of representations that are attached to distinct emotions (which reflect the quantity of drive energy, manifested in subjective experience through affective states). DMs (e.g., repression) operate on these representations so that the energy from the drive is free to be displaced onto other, non-threatening representations.

#### Melanie Klein's Views on Aggression

Contrary to Freud, the object relations theorists who followed shortly after gave aggression a central role in their models, seeing it as being in a reciprocal relationship with personality structures. Indeed, while DMs still contributed to modifying the manifestation of aggressive tendencies, the notion of degrees of maturity for the personality structures was introduced. This allowed for aggressive tendencies to be reduced upstream (i.e., top-down) *via* the transformation of negative thoughts and feelings into more nuanced, less intense ones. In addition, there was a gradual shift in the importance given to the social environment as a source of aggression. Finally, because early (immature) DMs during child development played a major role in the formation of self/other representations, their operations could distort the image of the self and others.

As the founder of object relations theory (a branch of psychoanalytic theory that focusses on the interpersonal efforts exerted by the child to obtain love, empathy, admiration and trust, as opposed to the Ego who tries to satisfy the drives in Freud's model), Melanie Klein (Klein, 1975a,b,c) followed Freud by using the concept of the death drive in her theory of aggression. While analyzing young children during therapy sessions, Klein observed primitive anxieties and phantasies that she interpreted as related to the drive and the workings of the unconscious mind. Contrary to Freud's view, she believed that the ego was present at birth and, through phantasies (mental representations), could represent all the pleasant or unpleasant somatic sensations coming from the life and death drives. She also believed that mental representations and affects (or energy) were not dissociable and that the Ego and the Id formed one single agency. As such, the fantasy life of infants is perceived as a real event and experienced in real physical sensations that are pleasant or unpleasant (e.g., feelings of hunger being experienced as a personal attack from hostile forces). Consequently, the motivation for implementing the DMs isn't the conflict between the Ego and the Id (as was the case for Freud), but rather between internalized images of the Ego and others (as objects). Also, unlike Freud's view of the drive as continually in search of a discharge of tension, Klein considered the drive as primarily relational, in search of objects. She believed that, in relational contexts with other objects (e.g., a primary caregiver or parent), the infant experienced the death drive as an intolerable fear of annihilation. To protect the infant, several DMs are set into motion. First, the infant splits both the ego and the object into more manageable parts by separating them into “good” and “bad” images. Because the infant's mind is still immature, it is unable to separate “the self” from “the object” and so, contradictory feelings such as gratification and deprivation (that stem from the Ego) are expelled and placed onto the object through a mechanism called *projection*. The object is then perceived as either good or bad, which creates a feeling of protection for the infant (from the good object), as opposed to anxieties of persecution (from the bad object). With the help of another mechanism called *introjection* (which is inseparable from projection), the infant now tries to place what is experienced as good (during the interaction with the mother) inside the self. This results in the infant feeling as though it possesses the good object. Bad objects may also be introjected into the self so that the infant can identify with them and control them (*projective identification*). As the good and bad objects are taken back into the infant's psyche (with the good objects needing protection from the bad ones), a new cycle of fantasies will follow.

In Klein's model, the goal of the death drive is the destruction of internalized objects that generate negative feelings such as envy and greed. Here, envy is both the desire to acquire the idealized aspects of the object and attack it (the object) because of the suffering caused by not possessing those idealized aspects. Greed, on the other hand, only desires to extract all the good aspects and ultimately results in the object's destruction. During development, the infant will go through a stage called paranoid-schizoid (briefly described above), followed by a stage of depression during which they are less concerned about protecting the self and more concerned (or worried) about the damage they may have caused to the object when acting out their aggressive phantasies. This sets the stage for several other transformations in the formation of their personality, allowing them to develop new capacities such as being able to tolerate ambivalent feelings, have more realistic perceptions of external objects, and begin to feel gratitude toward them. In parallel, the core of the Ego is formed by internalizing objects that are good, secure, and whole. Klein preferred to call these developmental stages “positions,” as she believed that—unlike stages, which are progressed through in somewhat of a linear fashion— positions represent ways of perceiving and relating that may oscillate throughout development and into adulthood (Klein, 1946). Therefore, Klein believed that, depending on the experiences of the child, these two positions (paranoid-schizoid and depressive positions) would persist later in life and characterize the nature of the affects and defenses that manifest in the relationships of the adult.

#### Kohut's View on Aggression

Heinz Kohut's psychology of the self (Kohut, 1966, 1971, 1972, 1977) rapidly became a new and influential strand in psychoanalysis and placed more emphasis on the personality structure known as the self. Although the *self* can be defined in several ways, object relations theorists primarily conceptualize it as an intrapsychic representation of the person as a whole entity. For Kohut, the self is the central agency in the structure of personality and represents the core of the individual's experiences, their initiatives and all their impressions. Unlike the Id described in classical psychoanalytic interpretations, Kohut proposed that the self was gradually accessed through the therapist's ability to empathically immerse themselves into the inner life of the patient. In his early writings, his work with patients who suffered from narcissistic problems (e.g., compulsive rage and aggressive behaviors) led him to revise Freud's theory of narcissism. For Freud, narcissism was one of the first stages in the development of the libido (sexual energy), wherein it is invested in the Ego as its first love object. Only during a subsequent stage does the libido detach itself from the Ego and then attach itself to external objects. Kohut, rather, made a distinction between the narcissistic libido and the object libido and suggested that they developed independently. Through interactions with the parent, the child initially develops a cohesive (archaic) self that separates into two types: a grandiose self that is all-powerful and demands admiration, and a perfect idealized object with which the self identifies. During this period, the parent is not perceived as a separate entity from the child's self, but rather as a self-object, one that assumes the responsibility of meeting the child's narcissistic needs. Kohut believed that, in healthy development, the parent responded empathically to these needs, and so the child's self would develop normally and ultimately take over the role of supplying the admiration and idealization that had initially been provided by the parent.

Although these needs are thought to persist throughout the person's life, the need for empathic support from (external) others to maintain self-esteem is thought to gradually decrease with age. At the same time, the child's perception of themselves and others becomes more realistic; their grandiose self slowly transforms into personal ambitions and the idealized object becomes their values and ideals. In the case that the parent is unable to empathically support the child's need for admiration or is inadequate in serving as a figure of admiration (often due to psychopathology), an archaic self will persist, be repressed, and come into conflict with the real ego (e.g., the reality-oriented structure of the ego). For the adult who persistently needs self-objects to be admired or continues to idealize people with whom they identify, the self remains in its primitive form. Since the person with an archaic self is “perfect”, and their surrounding environment is simply an extension of themselves, an environment that fails or frustrates the person (by not meeting narcissistic demands) will quickly threaten the self with the fear of disintegration (an anxiety resembling psychic death). According to Kohut, the person reacts to this humiliation and injury to self-esteem with “narcissistic rage,” a form of reactive aggression ranging from a simple annoyance to anger, to intense fury. The motivations underlying narcissistic rage are revenge, restoration of the person's sense of omnipotence, and absolute control over their environment. For Kohut, the purpose of DMs (e.g., idealization and omnipotence) is not to reduce anger or destructive behaviors, but rather to protect the individual's fragile self. As such, Kohut believed that narcissistic rage is under the service of the restoration of the archaic self.

### Defense Mechanisms and Aggression

While the relationship between inhibition processes and reactive aggression is relatively straightforward (i.e., inhibitory processes delay or suppress the aggressive response), it is comparatively more complex between DMs and aggression and may yield different results. The three psychoanalytic models presented above may be useful for understanding this relationship. In Freud's model, DMs can either increase or suppress aggressive impulses, or maintain their initial level. They can operate on the action, the object (i.e., the person), or the goal of the aggressive drive. For example, in the case of repression, an aggressive impulse that seeks satisfaction through a reduction of tension (with the help of the Ego) may be refused access to consciousness. This totally deprives the individual of the opportunity to deal with their psychological reality, and so it is as though the aggressive desire never existed. In the case of *reversal into its opposite*, the aggressive impulse can result from the transformation of a fear, as exemplified by the child who transforms their anxiety into aggressive behaviors by identifying with their aggressor (e.g., a parent, a teacher). When the defense acts on the object of the drive, aggressive desires that target the object will be displaced onto a less threatening object, leaving the pressure from the aggressive drive entirely. Finally, when the defense acts on the goal of the drive (as demonstrated with *sublimation*) aggressive desires can be satisfied by goals that are socially sanctioned and encouraged, leading to new and adaptive ways to express aggressive impulses.

In Klein's model, the DMs appearing most related to aggressive impulses are found in the paranoid-schizoid position (introjection, projection, splitting, projective identification). Here, both the drive seeking the object and the DMs are expressed through phantasy. To survive, the infant needs to possess (in their internal world) an image of a good/ideal object that will bring them love and security and with whom they can identify. In contrast, the bad object would evoke a feeling of persecution, and experiences of frustration are experienced as a type of sadistic attack on the good/ideal object by the persecutory object. To avoid this from occurring, the early ego makes use of various primitive DMs, such as introjection for the good parts, projection for the bad parts, or splitting the ego and the object into good and bad images to keep them as separate as possible. It may also use projection identification, in which the ego projects the bad split-part of the self and the bad internal object onto the external object, followed by introjection of the bad object that remains under its control. Each of these mechanisms results in distorting the image of the self and the object, which creates a vicious cycle between the defenses, persecutory anxiety, hatred, anger, and aggression. As revealed in clinical settings, all of these inferred processes have been described by Klein in her account of the infant during the first year of life and have also been reported in adult individuals functioning at the level of the paranoid-schizoid position (e.g., Joseph, 1983).

Out of the three psychoanalytic models we presented, Kohut's model is the only one without a theoretical formulation of a link between the DMs and the vicissitude of the aggressive drive. Rather, Kohut's model posits that the aggressive response toward the provocateur serves a defensive function in that it attempts to restore the archaic grandiose self. Accordingly, it is possible that (in certain situations) aggressive behaviors represent a DM in the form of *acting out*.

### Synthesis of the Theoretical Analysis Between Socio-Cognitive and Psychodynamic Models

The comparative analysis revealed various similarities and differences between socio-cognitive and psychodynamic conceptualizations of sources, motivations, and processes that influence aggression. Table 1 above provides a useful framework for organizing the key elements and drawing out theoretical comparisons. We have synthesized the main findings and will report them by starting with sources of aggression and ending with the consequences that lead to its occurrence.

**Table 1:** Comparisons between socio-cognitive and psychodynamics models on various dimensions of aggression.

	**Socio-cognitive models**	**Psychodynamic models**
External sources of aggression	- Social provocation - Non-social or aversive situational conditions	- Failure of the self-object to meet narcissistic demands (Kohut)
Internal sources of aggression	- Faulty social information processing (Crick and Dodge) - Highly accessible aggressive cognitive structures (e.g., schemas, scripts; Anderson, Bushman, and Wilkowski) - Negative affect (Anderson and Buschman) - High trait anger (Wilkowski)	- Aggressive drive (Freud) - Aggressive and persecutory phantasies (Klein) - Grandiose self (Kohut)
Motivations for aggression	- Protection against a threat or offense	- Discharge of tension (Freud) - Destruction of bad objects (Klein) - Vengeance and restoration of the grandiose self (Kohut)
Implementation of regulatory processes	- Conscious awareness of conflict between social expectations, prosocial personal values, and aggressive impulses	- Unconscious conflict between internal prohibitions and aggressive impulses
Characteristics of regulatory processes	- Adaptive and effortful resources for resolving interpersonal conflicts	- Adaptive or maladjusted compromises for achieving unconscious motivations
Consequences of failed inhibition	- Loss of control over cognitions	- Loss of control over unconscious impulses

A major focus in socio-cognitive models is on the interaction between the person and the situation during a social encounter. Relatedly, one of the main sources of aggression in these models consists of factors that are external to the individual. As briefly mentioned above, situational factors represent an important causal source of aggression and include any potentially triggering antecedents of the social situation, such as interpersonal provocations (e.g., insults, slurs, hand gestures, bullying; Berkowitz, 1993; Anderson and Bushman, 2002; Ireland and Archer, 2002) peer rejection or victimization (Crescioni and Baumeister, 2009) exposure to violence and aggressive cues (e.g., weapons; Carlson et al., 1990), as well aversive environments that cause physical or emotional discomfort (e.g., hot weather, loud noises, traffic). Similarly, Kohut's model places relational frustrations at the root of aggression, or more specifically (notably for individuals with a narcissistic personality disorder), a type of frustration that is perceived as a blow to self-esteem. In contrast, psychodynamic theorists such as Freud and Klein prioritized the existence of an aggressive drive, placing situational factors as secondary to internal causal factors. For them, situational factors only influenced the expression or the experience of the drive. For Freud, a person will simultaneously attempt to comply with the rules of the external reality (i.e., the real world) by controlling their aggressive drives and by asserting themselves against external threats. For Klein, while aggressive and persecutory phantasies about the world can manifest with or without an aggressive environment, actual experiences of aggression with real people confirm the impression that “the world is bad.”

When attempting to understand the source of aggression, both the socio-cognitive and psychodynamic models acknowledge that the situation alone cannot fully explain a person's behavior. Rather, the *interpretation* of the situation is unanimously pointed to as a primary determinant. In socio-cognitive models, this interpretation will depend on the way that social information is processed, which in turn will depend on the contents stored in the person's cognitive structures (e.g., schemas or scripts). Aggressive scripts (comprised of associated concepts stored in long-term memory) are thought to develop through past experiences and gain accessibility with frequent rehearsals in real-life situations. As such, a person who was repeatedly exposed to aggression early in life (e.g., *via* media violence, abusive parenting, etc.) is proposed to have highly accessible aggressive scripts that are carried over into many social situations and essentially “hijack” interpretive processes, resulting in a HAB. In psychodynamic models, the interpretation of the social situation is influenced by unconscious workings of the mind (i.e., domains of personality), and so the person is not in control of these processes. Rather, the pressure that these domains exert on the person's thoughts and feelings force them to develop defensive maneuvers to protect themselves from anxiety. Depending on the defensive strategies they use, the drive (Freud), the unconscious phantasies (Klein), or the archaic self (Kohut) may manifest as aggressive thoughts, emotions, or behaviors.

The causal contribution of emotions is also addressed in all the models, albeit to varying degrees of influence. All socio-cognitive models acknowledge that negative emotions, such as anger, affect the way a person perceives, interprets, and reacts to social events. While the SIP model remains vague about the impact of anger [when intense, it typically accompanies the act; Crick and Dodge (1996), p.1000], both the GAM and the ICM models consider it an important individual factor, exemplified in people who are highly prone to anger and who characteristically interpret the behaviors of others as a threat. However, the extent to which anger biases a person's thinking or increases their level of arousal isn't necessarily as stable as their modes of processing social information. In other words, socio-cognitive models posit that the degree to which anger (or person factors in general) motivates a person to respond with aggression is entirely dependent on its influence on their way of thinking. Also, the type of intense anger that would be needed to set an aggressive impulse into action is more likely a state factor associated with a personality trait. In psychodynamic models, the primary motivational influence stems from the drive, which is sometimes mistakenly assumed as also transient nature. The reason for this is because “hunger” is often used as a prototype for the drive, and since hunger can easily be satisfied with food, the drive could appear similarly quieted when its needs are met. However, the psychodynamic concept of the drive, as well as the unconscious phantasies and the grandiose self, are all described as having the potential to perpetuate their effects in the person's unconsciousness. More specifically, evoking an internal conflict (e.g., repressed aggressive drive, phantasy, or archaic self) can go undetected from the person's conscious awareness and still operate on mental processes throughout their life. Taken together, the overall difference between socio-cognitive and psychodynamic sources of aggression relates to the importance given to external and internal factors and whether aggression is conceptualization as a learnt behavior or an instinctive drive.

While the proposed motivations of aggression in socio-cognitive models are described differently in psychodynamic models, we find similar themes in almost all of them. For example, because socio-cognitive models characterize reactive aggression as a behavioral response to a perceived threat or offense, its principal motives are self-protection or an impulsive means to get revenge. Along a similar vein, Kohut's later theories posit that the goal of aggression is to protect the person's self-esteem, get revenge on the object that destroyed their illusion of omnipotence, and restore their grandiose self. Klein also described a need to protect, but for her, it was through phantasies of destruction to preserve good/ideal objects. Freud had a slightly different view from the others and thought that aggressive drives were motivated by the need for a discharge of tension through satisfaction. In some ways, however, the result of not satisfying these needs would likely be unbearable for the person, and so perhaps Freud's explanation concurs with the overall notion that aggression is motivated, in part, by the need to protect oneself.

Another similarity between socio-cognitive and psychodynamic models pertains to the assumed presence of a conflict between aggressive impulses and some form of resistance to the realization of these impulses. According to all accounts, it is *this* conflict that motivates the implementation of control/protective processes. Socio-cognitive models derive this conflict from social learning theory, in which people learn normative rules about aggression from external sources within their social environment (e.g., caregivers/parents, other authoritative figures, peers, etc.). They also learn and acquire prosocial values, which serve as guidelines for appropriate social behavior. When social situations become hostile or threatening (whether it is perceived as such or objectively the case), these social values will (ideally) motivate people to practice self-control by suppressing or delaying an aggressive impulse and finding alternative, non-hostile, ways to interpret the situation and/or clarify ambiguous social cues (i.e., cognitive reappraisals). This notion of a conflict between having a behavioral impulse and resisting the urge to act on it has effectively been operationalized in experimental settings using a stop-signal task (discussed below). The task involves having the participant perform a speeded choice reaction task (the “go” task) and occasionally withhold their response whenever the “go” stimulus is followed by a “stop” signal. Given that the participant must inhibit a response that has already been primed with the go stimulus, it is somewhat analogous to situations in which external cues trigger an automatic response, yet the appropriate response is *no* response at all. The inherent dilemma in this analogy is that to successfully stop a process that is already set in motion, the person must be able to recruit control resources at the conscious level. During social conflicts, socio-cognitive models propose that this possibility is strengthened by having personal values that prioritize interpersonal relationships. In psychodynamic models, the “conflict” that could potentially provide an opportunity for resisting or modifying an aggressive impulse happens entirely at the unconscious level. Also, these impulses manifest in unconscious desires and phantasies, and so it becomes difficult to describe counteracting forces from an objective point of view. For example, an individual might have feelings of guilt simply because they had an unconscious hostile thought toward a close relative. In psychodynamic models, prohibitions from the Superego and DMs could intervene in this type of conflict, but they do so without the intervention of human awareness.

Lastly, the way control processes are characterized in socio-cognitive models differs considerably from self-protective processes in psychodynamic models. While there are several proposed control processes in socio-cognitive theories, the ones most associated with models of aggression refer to intentional, effortful, and adaptive cognitive processes that operate to reduce hostile thoughts and inhibit aggressive behavioral responses. This usually encompasses any self-initiated cognitive process that interrupts automatic patterns of thought and allows the person to consider alternative ways to approach or understand the current situation. Examples include thinking about the negative consequences of the act, redirecting attention away from aggressive cues, or recognizing physiological indicators of anger and trying to “calm down.” To borrow Freud's metaphor, we could say that the Ego is attempting to mediate the situation and satisfy all the demands of the situation by finding an adequate solution to the problem. Evidently, if the person has deficits in processing social cues or is unable to control how they interpret the situation (possibly resulting from repeated activation of aggression-related associations in long-term memory), then this loss of cognitive control facilitates automatic cognitive processing and habitual response patterns will likely be repeated. In psychodynamic models, certain DMs are adaptative, while others are not. They are conceptualized as dynamic processes that, rather than being directed at the situation, are aimed at the individual for the purpose of protecting them from the distressing phenomena happening within them. There are several proposed DMs, each of which provides different solutions that produce different results regarding their impact on aggressive impulses. The compromise that lies between the defense and the drive represents the solution to the problem. However, if the defense fails to contain the unconscious desires, phantasies, or grandiose self, then the compromise may manifest as a symptom (e.g., the “horse phobia” of Little Hans, Freud's 5-year-old patient, representing his aggressive desires toward his father and projected onto horses). Throughout a person's life, new DMs can be added to the original ones, or even take their place. When the forbidden desires give rise to symptoms, it is as though the repression of the Ego has failed to keep them in the unconscious part of the mind and the individual has lost control of their instinctual life insofar as these desires will remain inaccessible or even accessible but in a disguised and non-recognizable manner (e.g., symptom) to consciousness, the part of the mind that would be able to deal with them in an adaptative way. In this sense, the psychodynamic explanation for failed inhibition differs from the socio-cognitive perspective, as deficits or overridden control functions are not a part of the underlying process.

## Part 2: Empirical Study on Control Processes and Defense Mechanisms that Influence Aggression

### Empirical Background

#### The Relationship Between Defense Mechanisms, Aggression, the Hostile Attribution Bias, and Response Inhibition

Since the beginning of its construction, the concept of DMs has grown in popularity and has been applied to other fields of psychology and psychiatry. Objective instruments were developed so that empirical studies in clinical and normal populations could attempt to distinguish defenses according to their level of maturity and adaptability (Gleser and Ihilevich, 1969). Stemming from these studies, a hierarchical classification of DMs was formed, the best known of which was created by Vaillant (1992) and consists of clustering the processes into four groups: psychotic, immature, neurotic, and mature defenses. In a longitudinal study among college men, Vaillant (1976) demonstrated that mature defenses correlated positively, and immature defenses correlated negatively with work and relationship success (Vaillant, 1976) and mental health (Vaillant, 1987). A common approach is to distinguish between less mature defenses, which involve distortion of self, others, or reality, and more mature defenses that contribute to effective functioning (Lingiardi and McWilliams, 2017). From a psychiatric perspective, the DSM-IV (American Psychiatric Association, 1994) defined DMs or coping styles as automatic psychological processes that protect the individual from anxiety or the perception of internal, or external dangers, and/or stressors. This latter definition combines two categories of processes have been contrasted by some authors: DMs—which are unconscious, unintentional, automatic, and rigid processes distorting the reality—and coping style, which are conscious, intentional, flexible, and adaptative processes (Hann, 1977). More recently, conceptualizations of DMs have deemphasized unacceptable thoughts and focused more on how DMs reduce negative affect and maintain self-esteem (Cooper, 1998).

There are many approaches for assessing DMs. Commonly used methods include applying rating scales to clinical interview transcripts (Perry, 1990) or material from projective instruments (Cramer, 1991), as well as using self-report questionnaires (Bond et al., 1983). Although these methods can be more vulnerable to response biases, self-report questionnaires are often used in empirical studies because they have several advantages. Apart from being inexpensive and simple to administer, results are easily collected without the need for interrater reliability. Another rationale for using self-report questionnaires stems from the hypothesis that, while DMs are unconscious processes, individuals who frequently use them can become conscious of their affective and behavioral derivatives and thereby rate themselves accordingly (Bond, 1992). One of the most cited self-report questionnaires in the research literature is the Defense Style Questionnaire (DSQ; Bond et al., 1983).

Most of the empirical studies that have examined the contribution of DMs in the expression of aggressive behaviors have primarily focussed on psychiatric populations. The first studies were conducted on auto-aggression (e.g., aggression toward oneself) behaviors among depressive patients. Results consistently showed that higher use of immature and image distorting styles and less use of mature style, as assessed with the DSQ-40 (a short version of the DSQ; see the description of the DSQ in Methods), were the best predictors of suicide attempts (Corruble et al., 2004; Hovanesian and Cervellione, 2009). When assessed individually, the DMs acting out, passive aggression, autistic fantasy and projection were shown to be used more often by “attempters” compared to “non-attempters” (Corruble et al., 2004). In a sample of borderline personality disorder patients with and without having had a suicide attempt, findings indicated that *splitting of other's image* was among the best predictors of suicide attempts (Lee et al., 2020). Koenigsberg et al. (2001) focused on the relationship between defense styles, as assessed with the DSQ, and impulsive aggression traits, as measured with two questionnaires. In a clinical sample with personality disorders, the researchers found that impulsive aggression was positively correlated with acting out and negatively correlated with both suppression and reaction formation.

Only recently have researchers begun to study the use of DMs among individuals who present difficulties with controlling their anger and aggression toward others. For example, Puhalla et al. (2016) compared individuals with intermittent explosive disorder (a DSM-5 disorder characterized by impulsive, anger-driven acts of aggression; American Psychiatric Association., 2013), a personality disorder comparison group, and healthy controls on the DSQ-40. Results showed that subjects with intermittent explosive disorder obtained higher scores than both comparison groups on immature defense styles and lower scores on mature style. Further, higher levels of acting out and lower levels of sublimation uniquely differentiated the groups. Data obtained exclusively with psychiatric populations have left open the question as to whether the relationship between DMs and the decision to act aggressively in a social situation also exists in non-clinical populations. If so, decreasing the use of maladaptive DMs for adults without psychopathology may be a potential target for prevention programs of aggression and integrative models of intervention for impulsive aggression (e.g., Gagnon et al., 2019). In support of this proposal, a recent study showed that the relationship between homophobia and aggression in university students was mediated by neurotic defenses (Set and Ergin, 2020).

While there are some similarities between maladaptive DMs and deficits in socio-cognitive processing (e.g., Cramer and Kelly, 2004; Whitman and Gottdiener, 2015), there is a paucity of research exploring this potentially significant association. For example, Cramer and Kelly (2004) compared adolescents with conduct disorder and adolescents with adjustment disorder on the use of DMs, as assessed with a projective instrument. Given evidence of deficits in socio-cognitive processing among delinquent and aggressive youths (e.g., see Crick and Dodge, 1994), and that certain cognitive biases appear similar to the DM projection (e.g., HAB), the researchers expected higher projection scores for the conduct disorder group, as compared to the adjustment group. However, results showed that the two groups did not differ on this variable. To make progress on this issue, it would be beneficial to make use of experimental methods that allow a more direct measure of HAB processes as they unfold in real-time. We believe that the implicit measure of hostile intent attribution (HIA) developed by Gagnon et al. (2016), the Hostile Expectancy Violation Paradigm (HEVP; see description in Methods), could serve this purpose.

Finally, the notion that a relationship exists between DMs and inhibitory control has been addressed in some way or another since the very beginning of psychoanalysis. In his “Project for a scientific psychology,” Freud (1895) theorized about the neural correlates of the inhibition exerted by the Ego on the experience of satisfaction. The similarity between inhibition and DMs such as repression has been the topic of a few contemporary essays as well (e.g., Erdelyi, 2006; Bazan, 2012). For example, Erdelyi (2006) discusses research on motivated (directed) forgetting as relevant to Freud's repression. Moreover, the interest in discovering possible neural substrates of psychodynamic concepts (e.g., DMs) has been revived by several neuroscientists who, due to recent technological advancements in neuroimaging, have led innovative experimental studies. Indeed, a series of studies (Shevrin et al., 1992, 1996, 2013) has been conducted, that made use of event-related potential markers of subliminal unconscious processes and demonstrated a functional relationship between unconscious inhibition (alpha power), unconscious conflict primes, and conscious symptom targets. Even though extant findings from dynamic neuroscience suggest a link between DMs and inhibitory control processes, the specific DMs involved have yet to be clearly identified.

#### Relationship Between Inhibitory Control, Aggressive Behavior, and Hostile Attribution Bias

Inhibitory control comprises various executive functions that enable a person to control their emotions, thoughts, and behaviors (Diamond, 2013), eliminate a strong propensity to act (Hsieh and Chen, 2017), or initiate and/or inhibit a behavioral response (Eisenberg et al., 2005). Importantly, inhibitory control is an umbrella term that includes several controlled processes, one of which is the prepotent response inhibition—the capacity to suppress a dominant (prepotent), automatic response (Friedman and Miyake, 2004; John and Gross, 2004; Kalisch et al., 2006).

The Stop Signal task (SST) is a paradigm that is often used to assess inhibitory control (Verbruggen and Logan, 2008). In the SST paradigm, as already mentioned before, the participant executes a go task, often by reporting the presence of a stimulus, and occasionally a stop signal follows the go stimulus, instructing the participant to withhold their response (Verbruggen and Logan, 2008). The SST can also be used to measure the latency of the stop process, or the Stop Signal Reaction Time (SSRT; see description in Methods), whereby faster reaction times index greater inhibitory control process (Verbruggen and Logan, 2008). As such, the SST could represent a relevant method for examining the relationship between response inhibition and reactive aggression.

Empirical attention has been drawn to associating deficits in inhibitory control processes with reactive (impulsive) aggressive behavior. A preliminary study using an SST task found that aggressive children showed weaker inhibitory control (probability of inhibition) and slower inhibitory processes (SSRT) compared to controls (Oosterlaan and Sergeant, 1996), which was suggested to reflect the idea that poor inhibitory control in aggressive children may relate to an overactive behavioral activation system. In a recent investigation, Hsieh and Chen (2017) used an SST paradigm to examine whether emotion regulation, in relation to inhibitory control, could predict aggressive behavior. Results showed that, for participants with low inhibitory control, there was a significant difference between high and low emotion regulation on aggressive behavior. Specifically, participants who had more difficulty with emotion regulation were more aggressive than participants better able to regulate their emotions, whereas this effect was not seen for participants with high inhibitory control. Relatedly, a previous study using an emotional version of the SST task examined response inhibition between individuals with high trait aggression and low trait aggression (Pawliczek et al., 2013). Their results showed a longer SSRT (indicating weaker response inhibition) in the high trait aggression group compared to the low trait aggression group. Taken together, the above findings support the idea of an inverse relationship between the capacity to control one's emotions and the tendency to act on aggressive reactions.

To our best knowledge, there is a paucity of studies investigating the putative relationship between inhibitory control and the HAB. In a study examining a related association, Choe et al. (2013) demonstrated that the level of effortful control among preschool children was negatively correlated with the HAB. Moreover, exploratory analyses indicated that level of effortful control moderated the effect of advanced “theory of mind” on the HAB. Hence, children with poorer effortful control may have more difficulty inhibiting hostile attributions (particularly when in ambiguous social contexts) than children with better effortful control (Choe et al., 2013). However, this study measured inhibitory capacities using a toddler-age behavioral battery and the HAB was assessed with hypothetical scenarios administered to the children. The N400 is a component of time-locked EEG signals known as event-related potentials (ERP) obtained *via* ERP study. The N400 amplitude (described in the next section) measured in the HEVP task is a good index of HAB (Gagnon et al., 2017), and to date, no studies appear to have used this technique to investigate the relationship between inhibitory control and HAB. To assess this relationship, it seems legitimate to make use of SST and N400 effect, given the fact that HABs are spontaneous inferences (Gagnon et al., 2016).

#### The Relationship Between the N400 Component and Aggressive Behavior

Event-related potentials (ERPs) reflect the change in electrical activity produced by the brain in response to an external stimulus or an internal (e.g., cognitive) event (Luck, 2014). The N400 is an ERP component that can be measured using electroencephalography (EEG) to investigate the underlying processes of social cognition (Bartholow et al., 2001; Kutas and Federmeier, 2011; Gagnon et al., 2016). For example, several studies have demonstrated that the N400 can be used as a valid index of social expectancy violations resulting from interpretation or attribution biases (Leuthold et al., 2012; Gagnon et al., 2016). By presenting participants with a first sentence that establishes a social context, followed by a target sentence ending with a critical word or a final sentence that clarifies the social situation described previously, the resulting amplitude of the N400 component allows researchers to assess for expectancy violations. Indeed, using this type of paradigm, Leuthold et al. (2012) found a larger N400 effect for critical words that were semantically inconsistent with expected word forms (related to feelings of the character, based on the context), as compared to critical words that were consistent.

A similar paradigm, the HEVP, was developed by Gagnon et al. (2016) and used to assess the neural events underlying the HIA and expectancy violations in aggressive and non-aggressive individuals. Participants were presented with social scenarios comprised of an initial sentence establishing a hostile or non-hostile context, followed by a character's ambiguous behavior, then a critical word that clarified the intention (hostile or non-hostile) underlying their behavior. When the critical word violated hostile expectations, a larger N400 response was found for aggressive compared to non-aggressive individuals. These findings were consistent with previous reports (Zelli et al., 1995; Wilkowski and Robinson, 2010), indicating that aggressive individuals show a tendency of attributing hostile intentions to the behaviors of others (particularly in ambiguous social contexts).

In a study by Stewart et al. (2010), the N400 was used to examine the association between anger styles and attentional biases to negatively valenced stimuli during an emotion-word Stroop task. Briefly, the anger styles assessed were acting-out, a propensity to express anger verbally or behaviorally toward people or objects, and acting-in, a propensity to suppress or inhibit outward signs of anger and/or withdraw from an anger-inducing context (Stewart et al., 2010). Results demonstrated that high anger-out individuals elicited a larger N400 effect to negative words compared to high anger-in individuals (Stewart et al., 2010). The researchers suggested that individuals who are highly aggressive require more attentional effort to distract themselves from negative information, especially if the information relates to anger. This assumption agrees with previous evidence demonstrating an association between cognitive capacities related to effortful control, trait anger, and level of aggressivity (Posner and Rothbart, 2000).

Other ERP components have also been used to assess aggression-related cognitive biases and confirm associations between high aggression and the HAB (see Godleski et al., 2010; Sun et al., 2020 for examples). The current study focusses on the N400 as it has previously been shown as a valid index of HABs among aggressive individuals and non-aggressive individuals (Gagnon et al., 2017) using the HEVP (Gagnon et al., 2016). Further, there is a vast literature converging on the idea that aggressive individuals may present deficits in inhibitory control processes that serve to regulate aggressive impulses, which has been empirically shown to predict individual differences in reactive aggressivity and HABs (Posner and Rothbart, 2000; Wilkowski and Robinson, 2008, 2010; Choe et al., 2013; van Adrichem et al., 2020).

#### Objectives

On the empirical level, our objective was to verify the associations between a neurophysiological measure of the HIA and a self-report measure of reactive aggression on the one hand, and two key concepts of processes that influence aggression according to socio-cognitive and psychodynamic models on the other hand: defense styles and prepotent response inhibition. Regarding the defense styles, given some evidence suggesting that individuals with an intermittent explosive disorder use immature DMs more often, and mature DMs less often (Puhalla et al., 2016), it was expected that reactive aggression scores among non-clinical participants would be positively correlated with image distorting scores, and negatively correlated with adaptative scores, as assessed with the DSQ-60. The HIA was measured with two indexes stemming from the hostile and non-hostile conditions (respectively) in the HEVP. In the non-hostile condition, a HIA tendency is reflected by a larger N400 effect (negative-going ERP deflection) following non-hostile critical words that violate expected hostile intent. In the hostile condition, a HIA tendency is reflected by a smaller N400 effect following hostile critical words that violate non-hostile expectations. Also, in light of documented deficits in socio-cognitive processing among aggressive youths that includes the HAB, and that this bias is functionally similar to certain immature DMs, such as projection (Cramer and Kelly, 2004), it was hypothesized that image distorting scores, as well as scores for related DMs such as projection, would be negatively correlated with the N400 effect in the non-hostile condition (violation of hostile intent expectations) and positively correlated with the N400 effect in the hostile condition (violation of non-hostile expectations).

Regarding response inhibition, given evidence suggesting that individuals with low effortful control capacity are less able to control their aggressivity (Liu et al., 2014; Verona and Bresin, 2015) and that inhibition deficits in response to angry faces are associated with trait aggression (see Denny and Siemer, 2012), it was expected that aggression scores would be positively correlated with SSRT scores, particularly during emotional (angry face) stop-signal trials. Also, given a previous finding suggesting that individuals with low effortful control are less able to inhibit a HIA in ambiguous social contexts (Choe et al., 2013), we expected SSRT scores to be negatively correlated with the N400 effect in the non-hostile condition, and positively correlated with the N400 effect in the hostile condition.

Finally, the last objective was to explore associations between defense styles and SSRT. Given the paucity of studies on DMs in relation to cognitive inhibitory processes, no hypothesis was made. Similarly, we explored associations between the individual DMs, reactive aggression, the HIA, and response inhibition.

### Methods

#### Participants

Participants were recruited by advertisements in the local community and university campuses as part of a prior study examining aggression and impulsivity (Gagnon and Jolicœur, 2014). A subset of this sample was used for the current analyses, which included 10 male (31.3%) and 22 female (68.8%) participants (mean age = 30.0 years, SD = 9.0; mean education = 16.0 years, SD = 3.7). All participants underwent an initial screening interview that included a brief description of the study, followed by a series of questions assessing sociodemographic information and study inclusion and exclusion criteria. Potential participants were informed that the study comprised two 60–90-mins test sessions, ~3 h in total. To be included in the study, participants had to be between 18 and 55 years old, be able to speak and read in French, and have normal or corrected vision. Respondents were excluded from participation if they had less than a 6th-grade level of education, reported a previous serious head injury, or had a history of psychosis. Before the visit to the laboratory, participants were asked to fill up the questionnaires (DSQ-60 et RPAQ; see below). Then, participants were invited to the laboratory and asked to refrain from alcohol consumption and recreational drug use for 12 h prior to each session. All participants provided written informed consent and received 40$ (CAD) at the end of their participation.

#### Assessments and Measures

##### Hostile Expectancy Violation Paradigm (HEVP)

The HEVP developed by Gagnon et al. (2016) was used to measure the HIA. It consisted of 320 scenarios depicting everyday social interactions. Participants were told to imagine the thoughts and feelings of the characters as if they were in the situation and had to understand why the person was behaving in such a manner. Each scenario contained two sentences that established the context as either hostile or non-hostile (first context sentence), during which a fictitious character behaved in an ambiguous manner toward the reader (second context sentence), followed by a third sentence ending with a target word that revealed the underlying intention (hostile or non-hostile) of the character's behavior (see Table 2 for examples). The condition of the scenario (hostile or non-hostile) was dependent on the nature of the target word (hostile or non-hostile). Each scenario was written in two similar versions that shared the same ambiguous behavior (second context sentence) and intention (target sentence) but differed regarding the first context sentence (i.e., one hostile version and one non-hostile version). For each scenario, the initial context (first context sentence) was either matched or mismatched with the character's intention (target word).

**Table 2:** Examples of four possible scenario sentences (translated from original French version).

**First context sentence**	**Second context sentence (ambiguous behavior)**	**Target sentence (intention)**	**Condition**
Non-hostile		Non-hostile	
Your colleague helps you to lose weight.	She brings cookies to work and doesn't offer any to you.	Your colleague does not want to *displease* you^*^.	Non-hostile/ match
Hostile		Non-hostile	
Your colleague is not nice to you.	She brings cookies to work and doesn't offer any to you.	Your colleague does not want to *displease* you.	Non-hostile/ mismatch
Hostile		Hostile	
In a bar, there is a stranger who likes to make fun of everyone.	He walks toward you.	The stranger wants to *insult* you^*^.	Hostile/ match
Non-hostile		Hostile	
In a bar, there is a stranger who likes to have a conversation with everyone.	He walks toward you.	The stranger wants to *insult* you.	Hostile/ mismatch

* *In French, the pronoun “you” precedes the verb, allowing for the target sentence to end with the critical word*.

Participants were tested in an electrically shielded booth with ambient light kept at a low level. Word stimuli were presented in white 14-point Helvetica font on a black background at the center of a 17-in. computer monitor at a viewing distance of 57 cm. Approximately three characters subtended 1o of visual angle. All participants completed four initial practice trials followed by 10 experimental blocks comprised of 17 trials (16 experimental scenarios and one filler scenario to ensure comprehension), divided by short breaks, the duration of which was determined by the participant. For each trial, participants were asked to read the first two sentences (i.e., initial context and ambiguous behavior) that were presented on the screen for a minimum duration of 1,500 ms and press the space bar once they had completed reading. Following this, a blank screen appeared for a duration of 500 ms, after which participants fixated a crosshair at the center of the screen for a duration of 1,000 ms. They were then presented with the third sentence (revealing intention) and asked to maintain fixation at the center of the screen as each word was displayed centrally for 300 ms, separated by 200 ms blank intervals. Following a 2,000 ms interval during which participants again fixated a crosshair, the next sentence (context) was displayed. The filler scenario was included among the 17 trials in each block and followed by a true or false comprehension question, which resulted in a mean correct response rate of 88%.

##### Stop-Signal Task (SST, Rebetez et al., 2015)

After the ERP procedures, participants were administered the Stop-signal Task (SST). The version of the SST task used in the current study had participants view images of facial expressions (male and female) displaying neutral or angry emotion. The images were taken from the Karolinska Directed Emotional Faces (KDEF), a validated standardized database of facial expression images varying in emotional content (Goeleven et al., 2008). Participants were instructed to differentiate the gender of the facial images by pressing “C” for a female face and “N” for a male face as quickly and accurately as possible and to inhibit their response if a “stop signal” appeared. In a stop trial, a beep sound served as stop-signals appearing a few milliseconds after the appearance of an image.

The task was constructed using the E-Prime package (PSS version E-Prime 2.0) and consisted of four blocks (2 neutral and 2 angry) of 64 trials each, for a total of 256 trials, 25% of which were stop trials. To avoid potential order effects, participants were randomly assigned to one of two versions of the task in which the order of the emotional content across the four blocks was counterbalanced. The first stop-signal delay (SSD)—the time interval between an image presentation and the “stop” signal—was set at 250 ms and subsequent SSDs were determined based on a tracking procedure: if the participant successfully stopped a response during a trial, the subsequent SSD increased by 50 ms; if they were unsuccessful, it decreased by 50 ms (Kalanthroff et al., 2013). The following outcome variables were considered for both conditions (angry and neutral): mean reaction time (MRT), non-response rate, categorization error rate, and stop-signal reaction time (SSRT). The SSRT represents the time it takes for the participant to inhibit a dominant response on 50% of the trials, and so it is thought to reflect a person's inhibitory capacity. Given that no button is pressed during a successful stop trial, the SSRT is an estimation and was calculated by subtracting the mean SSD (using the tracking procedure and 50% probability of response inhibition) from the n^th^ percentile of MRTs corresponding to the percentage of errors (Verbruggen et al., 2008). Only the SSRT was used in the present study.

##### Defense Style Questionnaire (DSQ-60)

In its first version, the DSQ was composed of 88 items used to assess 24 DMs (Bond et al., 1983). Factor analysis yielded four clusters of defenses, labeled defense styles, conceptualized in a hierarchy from immature to mature level of ego defense: immature, image distorting, neurotic, and mature. Over time, different versions of the DSQ were developed in an effort to improve reliability and validity, such as the DSQ-40, which is a short version composed of 40 items (Andrews et al., 1993). The DSQ-60 version is a 60-item self-report questionnaire used to measure the explicit derivatives of 30 DMs (two items per DM), based on the DSM-IV (Thygesen et al., 2008). Items are rated on a 9-point Likert-type scale ranging from 1 (“not at all applicable to me” to 9 (“completely applicable to me”) and participants were asked to indicate the degree to which they correspond to each of the 60 statements. The DSQ-60 measures three distinct defensive styles: “image distorting,” “affect regulating,” and “adaptive.” The DMs belonging to the image distorting style are splitting of self and others, projection, projective identification, help-rejecting complaining, and reaction formation. The affect regulating style comprises intellectualization, dissociation, isolation, and fantasy. Finally, adaptive defense—which is considered the most mature defensive style—comprises self-observation, humor, anticipation, and self-assertion (for a description of each of these defense mechanisms, see Perry, 1990). Scores for each style are calculated by taking the mean of the two items for each DM and adding them to the total mean score for each defensive style. The three defensive styles were determined through exploratory and confirmatory analyses by Thygesen et al. (2008) among English and French-speaking university students. Internal consistencies for the image distorting and adaptive defensive styles have been moderate (as = 0.64 and 0.61, respectively), with slightly more satisfactory results for the affect regulating style (α = 0.72) (Petraglia et al., 2009). In the present study, internal consistency coefficients were adequate for image distorting (α = 0.738), affect regulating (α = 0.772) and adaptive (α = 0.749).

##### Reactive-Proactive Aggression Questionnaire (RPAQ)

The current study used the French translated version of the RPAQ, developed by Gagnon and Rochat (2017). The RPAQ is a 23-item self-report measure, comprising 2 subscales that assess reactive and proactive aggression, respectively (Raine et al., 2006). The reactive aggression subscale contains 11 items (e.g., “How often have you reacted angrily when provoked by others”) and the proactive subscale contains 12 items (e.g., “How often have you threatened and bullied someone”). Items are rated on a 3-point Likert-type scale, ranging from 0 (“never”) to 2 (“often”), with higher scores indicating greater level of aggression. Reliability and validity of the scales have been tested in a variety of samples, including individuals convicted and incarcerated for criminal behavior, as well as non-clinical, healthy individuals, adolescents, undergraduate students, and adults from ages 6 to 64 years (Raine et al., 2006). In the present study, only Reactive aggression subscale of the RPAQ (RPAQre) was used and its internal consistency was adequate (α = 0.778).

#### EEG Recording

The electroencephalogram (EEG) data was recorded using 64 Ag/AgCl electrodes (BioSemi Active Two system) mounted on an elastic cap according to the International 10–10 System Acharya et al. (2016) and referenced to the average of the left and right mastoids. Horizontal eye movements were monitored using a horizontal electrooculogram (HEOG) and measured as the voltage difference between electrodes placed laterally to the external canthi. Eye blinks were monitored using a vertical electrooculogram (VEOG) and measured as the voltage difference between the two electrodes placed above and below the left eye. The EEG and EOG signals were recorded with a lowpass filter of 134 Hz at a sampling rate of 512 Hz. A high-pass filter at 0.01 Hz and a low-pass filter at 30 Hz were applied offline. Epochs from 200 ms preceding target onset to 1,000 ms after target onset were selected and baseline-corrected using the mean from −200 ms to 0 ms, relative to target onset. Ocular artifact reduction and data correction were both achieved with an independent component analysis (ICA) statistical procedure (see Drisdelle et al., 2017). Trials containing eye blinks (i.e., VEOG>50 μV within a 150 ms interval) and large horizontal eye movements (i.e., HEOG > 35 μV within a 300 ms interval) were excluded. Trials with EEG deflections greater than 100 μV during the (pre-selected) segmentation window on one or more of the 64 electrodes were further analyzed. If seven or fewer channels were detected in any given trial, they were interpolated from neighboring channels using a spherical spline interpolation. Trials containing more than seven channels with artifacts were rejected.

#### Statistical Analysis

All statistical analyses were performed using Statistical Package for Social Sciences Software (IBM SPSS Statistics 26). All 32 participants were included in the analysis. First, explorative analyses were performed using visual inspection of skewness and kurtosis parameters to verify the normality distribution assumption. To assess normality distribution, we performed Shapiro-Wilks test on all variables to be sure to detect all departures from normality. All the variables were normally distributed, except for the HN400RC effect. The HN400RC value of one participant was adjusted because it was an extreme value and thus undermined the normality of the distribution. The outlier's value has been replaced by 1 unit less than the adjacent lower value. The reaction time of the STT task was corrected when the standard deviation was greater than 2.

#### ERP Analysis

The data used in this study was originally analyzed by Gagnon et al. (2017). Among the original sample (*n* = 87), the 32 participants who completed the DSQ-60 were included in the present study. To verify if the present sample still demonstrates the N400 effects originally found in Gagnon et al. (2017), all EEG data analyses were reconducted using MATLAB with functions from the EEGLAB (Delorme and Makeig, 2004) and ERPLAB (Lopez-Calderon and Luck, 2014) toolboxes. Average ERPs were calculated per participant and condition (hostile-match, hostile-mismatch, non-hostile-match, non-hostile-mismatch) and time-locked to target word onset. A visual inspection of the global ERP revealed a significant negative deflection in the waveform between 450 and 650 ms for the non-hostile condition, which is within the standard N400 window (Kutas and Federmeier, 2011). ERP amplitude data at midline electrodes were analyzed separately from data recorded over lateral sites. These latter electrodes were pooled into six regions according to left-right and anterior-to-posterior dimensions. The three regions over the left hemisphere were defined as follows: left-anterior (AF3, AF7, F1, F3, F5, F7, FT7, FC1, FC3, FC5), left-central (TP7, T7 C1, C3, C5, CP1, CP3, CP5) and left-posterior (P1, P3, P5, P7, PO3, PO7, O1.) Three regions were defined for the homolog electrodes over the right hemisphere. Statistical analyses were performed by means of Huynh-Feldt corrected repeated measures ANOVA with variables Hostility (hostile, non-hostile), Consistency (match, mismatch), Hemisphere (left, right), and Location (anterior, central, posterior). The midlines electrodes were submitted to an ANOVA with variables Hostility, Consistency, and Location (anterior: AFZ, Fz, FCz; central: Cz, CPz; posterior: Pz, POz, Oz).

#### Correlations

To establish the association between the DMs and our measures of RPAQre, N400 effect, and SSRT, a correlation matrix was performed, in which correlations involving DMs are reported in Table 3. The level of significance was two-tailed.

**Table 3:** Correlations among defense mechanisms, reactive aggression total score, N400 effect and stop signal reaction time scores.

**DMs**	**RPAQre**	**NHN400RC**	**HN400RC**	**SSRT neutral**	**SSRT angry**	**SSRT total**
Image distorting	0.692^**^	−0.398^*^	0.395^*^	0.025	0.158	0.094
Affect regulating	0.241	−0.034	0.217	0.114	0.265	0.195
Adaptive defense	−0.423^*^	0.417^*^	0.031	0.089	−0.037	0.026
Acting out	0.790^**^	−0.401^*^	0.483^**^	0.111	0.185	0.152
Affiliation	−0.167	−0.004	0.192	0.039	−0.037	0.001
Altruism	−0.177	0.029	0.221	−0.081	−0.085	−0.085
Anticipation	−0.195	0.212	0.120	0.396^*^	0.257	0.333
Denial	0.294	0.004	0.307	0.069	0.153	0.114
Devaluation other	0.171	−0.130	0.102	0.083	0.195	0.143
Devaluation self	0.659^**^	−0.498^**^	0.323	0.065	0.262	0.168
Displacement	0.100	−0.064	−0.035	−0.095	−0.022	−0.059
Dissociation	0.499^**^	−0.285	0.329	0.012	0.134	0.075
Fantasy	0.306	−0.178	0.279	−0.005	0.149	0.074
Help rejecting complaining	0.634^**^	−0.426^*^	0.242	−0.037	0.078	0.021
Humor	−0.454^**^	0.221	−0.132	0.007	−0.019	−0.006
Idealization	−0.360^*^	0.304	−0.338	−0.129	−0.035	−0.083
Intellectualization	−0.065	0.155	0.039	0.143	0.223	0.187
Isolation	0.032	0.168	0.029	0.183	0.283	0.239
Omnipotence	0.125	0.149	0.349	0.354^*^	0.352^*^	0.361^*^
Passive aggressive	0.222	−0.331	0.127	−0.346	−0.339	−0.350^*^
Projection	0.498^**^	−0.416^*^	0.270	0.002	0.140	0.073
Projective identification	0.306	−0.145	0.445^*^	0.151	0.156	0.157
Rationalization	0.034	0.094	0.054	−0.189	−0.143	−0.169
Reaction formation	0.094	−0.140	0.169	0.055	0.140	0.100
Repression	0.353^*^	−0.118	−0.109	−0.140	−0.137	−0.142
Self-assertion	−0.367^*^	0.403^*^	0.049	0.018	−0.139	−0.062
Self-observation	−0.281	0.193	−0.050	−0.126	−0.175	−0.154
Splitting other	0.506^**^	−0.201	0.196	−0.020	0.080	0.031
Splitting self	0.579^**^	−0.265	0.349	0.025	0.143	0.086
Sublimation	−0.201	0.367^*^	0.090	0.041	−0.030	0.006
Suppression	−0.439^*^	0.364^*^	−0.105	0.079	0.120	0.102
Undoing	0.276	0.006	0.321	−0.015	0.074	0.030
Withdrawal	0.014	0.041	−0.066	0.076	0.152	0.117

*N = 32*.

* *Correlation significant at 0.05 (two-tailed)*.

** *Correlation significant at 0.01 (two-tailed). RPAQre, Reactive Proactive Aggression Questionnaire Total Score; NHN400RC, Non-Hostile N400 Right Central; HN400RC, Hostile N400 Right Central; SSRT Neutral, Stop Signal Reaction Time Neutral Face Condition; SSRT Angry, Stop Signal Reaction Time Angry Face Condition; SSRT Total, Total Stop Signal Reaction Time Neutral and Angry Face Condition*.

## Results

### EEG Data on the N400 Effects

**Figure 1:**
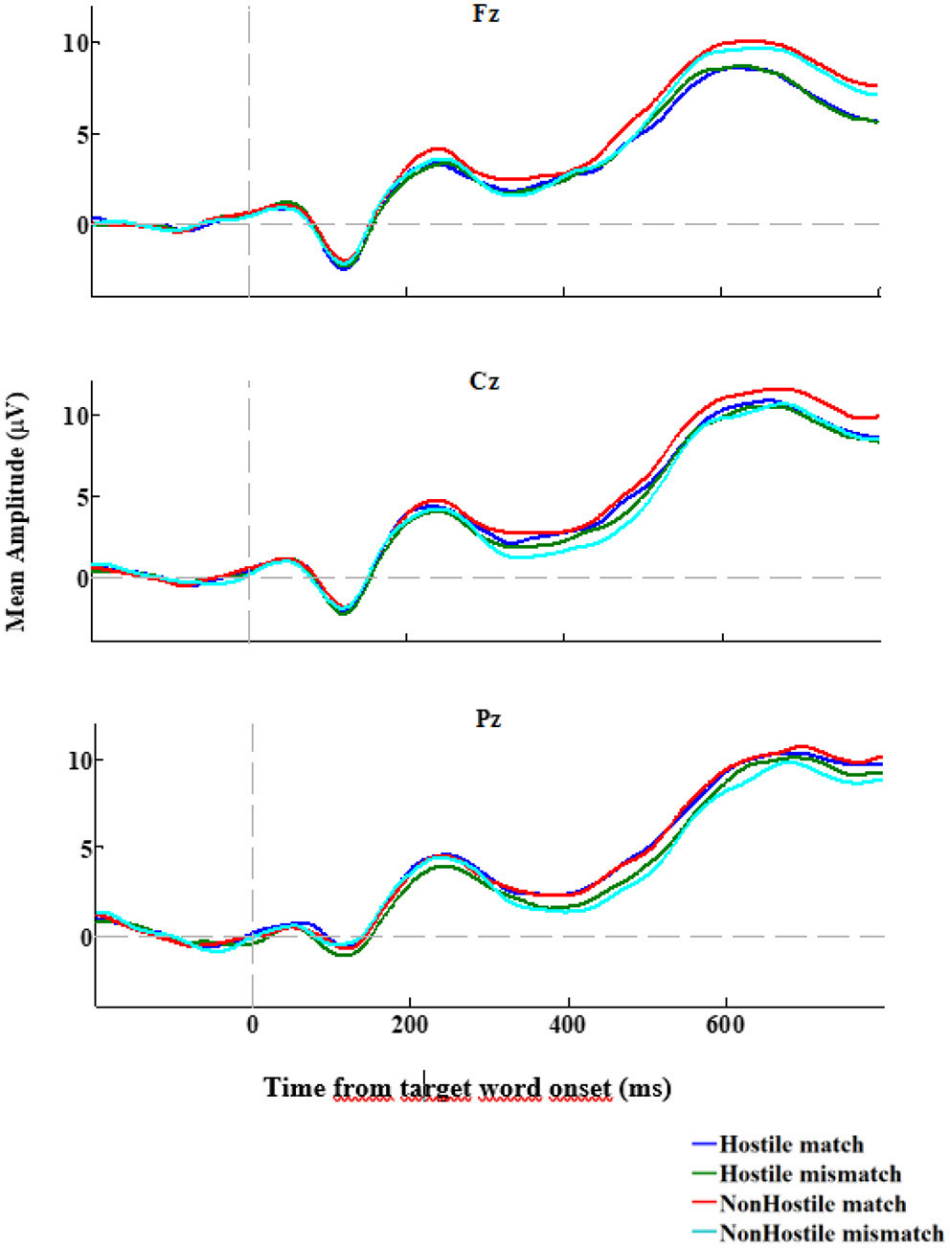
ERP waveforms at electrodes of anterior, central and posterior sites of the midline for the match and mismatch, and hostile and non-hostile condition (blue: hostile-match; green: hostile-mismatch; red: non-hostile-mismatch; aqua: non-hostile-mismatch).

**Figure 2:**
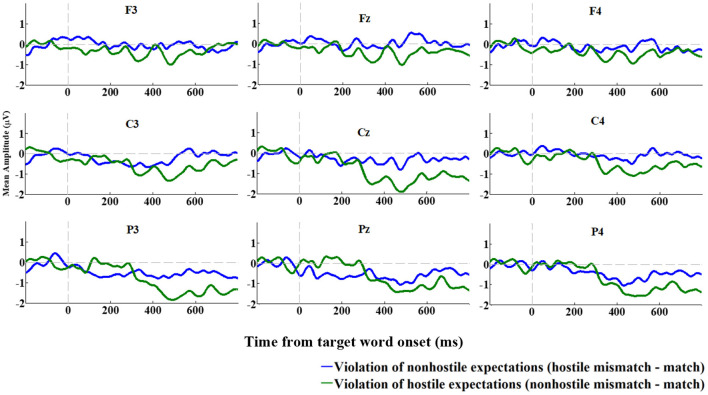
Mean ERP difference waveforms (ERP mismatch minus ERP match) for the hostile and non-hostile condition (blue: hostile; green: non-hostile).

**Figure 3:**
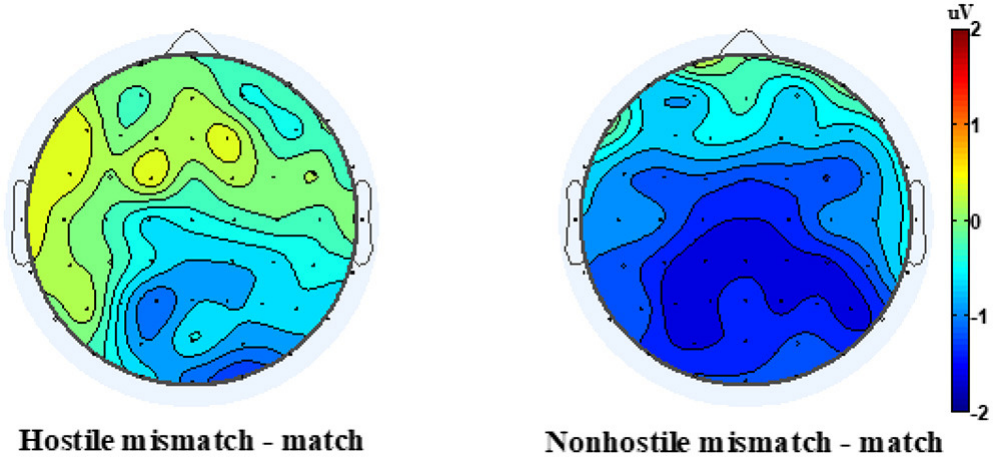
Topographic maps of mean ERP differences waveforms (ERP mismatch minus ERP match) for the violation of non-hostile intention expectency (hostile mismatch – match) and the violation of hostile intention expectency (non-hostile mismatch – match) conditions for the 450–650 ms post critical word time window.

Figure 1 presents the mean ERP waveforms at the six regions and the midline for the match and mismatch, and hostile and non-hostile conditions. The mean ERP difference waveforms (ERP mismatch minus ERP match) for the hostile and non-hostile conditions are presented in Figure 2, and Figure 3 displays the topographic voltage maps (mean amplitude from 440 to 650 ms relative to the onset of the critical word). Since we were interested in the impact of a violation of intention expectancies, we looked at the main effects of the ANOVAs and at interactions involving the effect of the Consistency factor. For the six regions ANOVA, we found a significant main effect for Consistency, *F*_(1,31)_ = 10.23, *p* < 0.003, indicating that the waveforms were more negative for the for the mismatch condition than for the match condition underlying the presence of the N400. There was also a main effect of Location, *F*_(2,30)_ = 4.18, *p* < 0.041. There was also a significant interaction between the factors of Hostility and Consistency, *F*_(1,31)_ = 4.62, *p* < 0.040. In the non-hostile condition, the waveform was significantly more negative in the mismatch condition than in the match condition, *F*_(1,31)_ = 16.59, *p* < 0.000, confirming the presence of the N400 (see Figures 2, 3), but there was no difference between match and mismatch in the hostile condition, *F*_(1,31)_ = 0.13, *p* = 0.72. Finally, there was a significant Consistency x Location interaction, *F*_(2,30)_ = 11.72, *p* < 0.000. The waveforms were significantly more negative in the mismatch condition than in the match condition at the central, *F*_(1,31)_ = 9.07, *p* < 0.005, and posterior, *F*_(1,31)_ = 19.68, *p* < 0.000 regions. There was no effect of Consistency in the anterior region, *F*_(1,31)_ = 0.74, *p* = 0.40. There was no other main effect nor interaction with the factor of Consistency.

The midline electrodes ANOVA showed main effects of Consistency, *F*_(1,31)_ = 11.22, *p* < 0.002, and Location, *F*_(2,30)_ = 16.03, *p* < 0.000. There was no other significant main effect. Finally, there was a significant Consistency x Location interaction, *F*_(2,30)_ = 7.35, *p* < 0.003. The waveforms were more negative in the mismatch condition than in the match condition for the central, *F*_(1,31)_ = 12.16, *p* < 0.002, and posterior electrode, *F*_(1,31)_ = 18.66, *p* < 0.000, underlining the presence of an N400. However, there was no effect of Consistency at the anterior electrodes, *F*_(1,31)_ = 0.57, *p* = 0.46. In sum, lateral and midline electrodes analysis showed the presence of the N400 when there is a mismatch between the expected intention and the intention from the target word, and that the component was larger in the non-hostile condition than in the hostile condition (for lateral electrodes), and larger over central and posterior than anterior sites.

### Data on Correlations Between All Measures

Descriptive statistics were computed on our behavioral variables of interest and among all our participants (*n* = *32*) to obtain the mean (M) as a measure of central distribution and the standard deviation (SD) as a measure of variability. More precisely, it was calculated on RPAQre (*M* = *8.625, SD* = *3.740*), on image distorting style (*M* = *1.522, SD* = *0.539*), affect regulating style (*M* = *1.908, SD* = *0.749*), and adaptive style (*M* = *3.244, SD* = *0.531*). In addition, it was calculated on SSRT Neutral (*M* = *315.017, SD* = *76.959*), SSRT Angry (*M* = *316.130, SD* = *78.214*) and SSRT Total (*M* = *315.573, SD* = *75.891*).

Correlation analyses were computed between all variables of interest and are reported in the correlation matrix (correlations not found in Table 3 are reported in the following text). Our analyses revealed a strong significant positive correlation between RPAQre and image distorting style. To a lesser extent, RPAQre was moderately negatively correlated with adaptive style, but not significantly correlated with affect regulating style. RPAQre was strongly negatively associated with the NHN400RC effect (*r* = −*0.526, p* = *0.002, two-tailed*) and moderately positively associated with HN400RC effect (*r* = *0.465, p* = *0.007*). Furthermore, the correlations were not significant between RPAQre and SSRT Neutral (*r* = *0.190, p* = *0.298*), SSRT Angry (*r* = *0.263, p* = *0.146*) and SSRT Total (*r* = *0.232, p* = *0.202*).

In addition, image distorting style was moderately negatively associated with NHN400RC effect, moderately positively associated with HN400RC effect, and not correlated with SSRT Neutral, SSRT Angry and SSRT Total. Affect regulating style was not significantly associated with NHN400RC effect, nor with HN400RC effect, nor with SSRT Neutral, SSRT Angry and SSRT Total. Adaptive style was moderately positively correlated with NHN400RC effect but not associated with HN400RC, nor with SSRT Neutral, SSRT Angry and SSRT Total.

Finally, NHN400RC effect did not correlate with SSRT Neutral (*r* = *0.310, p* = *0.085*), SSRT Angry (*r* = *0.249, p* = *0.169*) and SSRT Total (*r* = *0.285, p* = *0.113*). Likewise, HN400RC effect did not correlate with SSRT Neutral (*r* = *0.278, p* = *0.124*), SSRT Angry (*r* = *0.267, p* = *0.140*) and SSRT Total (*r* = *0.278, p* = *0.123*).

## Discussion

Several associations were hypothesized between the HIA (as indexed by N400 effects) and reactive aggression, as well as between the defense styles and prepotent response inhibition. Overall, the findings largely supported these hypotheses and provide support for the development of integrative operationalizations of aggression-related variables.

Concerning the defense styles, as expected, reactive aggression was strongly positively correlated with image distorting, and moderately negatively correlated with adaptative. This finding suggests defense styles play an important role in the regulation of aggressive behavior in non-clinical samples and extends previous research on defensive functioning among clinical populations. For example, as previously mentioned, the study by Puhalla et al. (2016) showed that participants with intermittent explosive disorder used immature defense styles more often and mature styles less often, as compared to participants with defense patterns comparative to healthy controls. The authors used the DSQ-40 version to categorize the DMs into three styles: mature, immature, and neurotic. The factor structure was slightly different from the one used in the current study (using the DSQ-60). Of the five DMs comprising the image distorting style, the immature style included splitting and projection. The mature defense style shared humor, sublimation, and anticipation, with the adaptive style. Despite discrepancies between various versions of the DSQ regarding how the DMs are clustered, our results converged with those of Puhalla et al. (2016). Indeed, a pattern of maladaptive behaviors, approaches for resolving conflicts, and regulating emotions, are all associated with increased use of immature defenses for aggressive individuals in clinical populations (Puhalla et al., 2016). This may stem from the distortions of reality caused by immature defenses to protect the self's integrity and result in deteriorating interpersonal relationships (Set and Ergin, 2020). Alternatively, the effects revealed by *less* use of mature defenses converge on the idea that aggressive individuals have difficulties in suppressing inappropriate impulses and thus are unable to convert them into more socially acceptable behaviors (Puhalla et al., 2016). According to Puhalla et al. (2016), the deficits in anger control and emotion regulation exhibited in aggressive individuals may impair their ability to respond with DMs that are more mature. To a lesser degree, related prepotent response inhibition deficits may also contribute to the defense styles used among non-clinical aggressive individuals. However, another possibility is that the contribution of defense styles on aggression among non-clinical individuals is independent of individual differences in prepotent response inhibition. Studies with larger samples would be able to verify the unique contribution of specific regulatory processes in explaining aggressive behaviors among non-clinical populations.

Regarding the HIA, as expected, image distorting was moderately negatively correlated with the N400 effect in the non-hostile condition (violation of hostile intent expectations) and moderately positively correlated with the N400 effect in the hostile condition (violation of non-hostile expectations). To our knowledge, this is the first study to show an association between the HIA bias and DMs that distort the image of self and others. A study by Cramer and Kelly (2004) examining a similar association was not able to demonstrate that adolescents with a hostile attributional bias differed in the use of projection, an image distorting DM. ERP techniques may be better able to measure spontaneous attributional processes (Gagnon et al., 2016). Moreover, our analyses showed that the adaptative style was moderately positively correlated with the N400 effect in the non-hostile condition. These findings suggest that image distorting DMs may contribute to a biased, maladaptive interpretation of the behavior of others, whereas adaptative DMs are related to stronger regulatory processes that protect the individual from faulty processing of social cues. This finding also underscores the importance of further developing integrated models of aggression to explain how these processes interact with each other. For example, using our proposed continuum framework, it is possible to conceptualize both image distorting style and the hostile attributional bias as occurring simultaneously in the development of hostile thoughts, which increase of negative affect (e.g., anger) and the likelihood of aggression.

Regarding prepotent response inhibition, contrary to what was expected, SSRT, in both emotional and neutral stop-signal trials, was not significantly correlated with reactive aggression. This finding diverges from previous studies showing that difficulties with inhibiting information within emotional contexts are significant predictors of aggressive behaviors (Denny and Siemer, 2012; Pawliczek et al., 2013; Liu et al., 2014; Verona and Bresin, 2015). One possibility is that the emotional stimuli (angry faces) used in the current study were not able to induce a strong enough emotional reaction to impair participants' reaction time. In support of this interpretation, a previous study showed that high arousal pictures that attract attention interfered more with responding and stopping than low-arousing pictures, whereas the valence of the pictures had little or no effect (Verbruggen and De Houwer, 2007). Similarly, given evidence showing that inhibitory control processes contribute to reducing hostile thoughts among non-aggressive individuals (Choe et al., 2013), it was expected that SSRT would be associated with the N400 effect in the HEVP. This hypothesis was not supported by our data. Few studies have verified the association between response inhibition and HIA bias. As previously mentioned, Choe et al. (2013) showed that individuals with low effortful control capacity, such as inhibitory control, might be less able to inhibit a HIA in ambiguous social contexts (Choe et al., 2013). However, hostile attributions of intent may be associated with factors other than inhibitory processes in driving aggressive behavior. For example, Arsenio et al. (2009) showed that attentional problems mediate the association between the hostile attributional bias and aggression among adolescents. According to the authors, *only* when the individual has difficulties paying attention to social cues do they become susceptible to feelings of frustration and to misinterpreting the intention of their peers. To our knowledge, this is also the first study to examine the association between response inhibition and the HIA. Our findings indicated a trend toward this association, as seen between the SSRT in the neutral condition of the SST and the N400 effect in the non-hostile condition of the HEVP (*r* = 0.310; *p* = 0.85), perhaps resulting from lack of statistical power due to the small size of the sample. Furthermore, to capture meaningful relationships between the HAB and prepotent response inhibition, it may be necessary that the sample comprises a sufficient portion of high trait aggression participants, a condition that was likely not met with our sample.

The last objective was exploratory in nature and no hypotheses were made regarding the associations between defense styles and SSRT, DMs and aggression, or HIA and response inhibition. First, defense style was not shown to be significantly correlated with the SSRT. However, certain individual DMs were significantly correlated with this inhibition index. Specifically, longer SSRT (weaker inhibition capacity) was positively correlated with anticipation and omnipotence, and negatively correlated with passive aggression. These findings suggest that mature defenses (anticipation) are not necessarily correlated with stronger inhibition capacities, whereas defenses that aim at controlling manifestations of aggression (passive-aggression) may relate to stronger prepotent response inhibition capacities. Alternatively, DMs characterized by phantasies of having unlimited power and potential (omnipotence) may relate to weaker inhibition. Given that the manner with which the defense styles cluster defensive processes risks eclipsing meaningful associations with other variables some psychodynamic authors have expressed doubts about grouping together such a great diversity of DMs with unique functions Laplanche and Pontalis, 2018), the present data suggests that examining associations at the level of individual DMs may be a better research strategy. It is noteworthy to underline the strong association found between acting out and the reactive aggression scores. This finding is consistent with previous research showing similar associations, supporting the theory that, for impulsive individuals, normal inhibition is bypassed into immediate action without regard of the consequences (Koenigsberg et al., 2001; Puhalla et al., 2016). However, Kohut's models suggests that aggressive behaviors may occasionally represent a DM (in the form of “acting out”), serving to restore the grandiose self after a narcissistic injury. Importantly, because DMs in the DSQ-60 are measured based solely on two items, these results must be interpreted with caution. Future studies exploring these relationships would benefit from using self-reports in conjunction with objective measures of DMs.

## General Discussion

This article serves a twofold purpose. In part 1, it bridges the gap between socio-cognitive and psychodynamic concepts of psychological processes that influence aggression and, by exploring various associations between them, advances our understanding of factors underlying aggressive behaviors. Using a theoretical approach, we identified similarities and differences by reviewing three prominent models from each perspective and comparing their respective conceptualizations of sources, motivations, and regulatory processes of aggression.

Our comparisons between socio-cognitive and psychodynamic models of aggression allowed us to identify certain themes that resonated across most of the models, as well as distinguish apparent conceptual differences.

Each of the models converged on three main ideas. First, both perspectives emphasize the importance of the manner with which a person interprets the social event and considers this factor a critical determinant in the emergence of hostility and aggression. They also share in proposing that the underlying motivation for reactive forms of aggression is either for self-protection or related to vengeance. Lastly, each describes a type of conflict or tension existing between two opposing forces—one that supports aggression and one that condones it—and acting as an antecedent for implementation of control/protective processes.

Our comparisons also revealed important theoretical differences. Proposed sources of aggression in socio-cognitive models place an important emphasis on the interaction between situational influences and maladaptive cognitive factors. Psychodynamic models prioritize the role of unconscious processes that can influence perceived environmental influences. While all the models (socio-cognitive and psychodynamic) acknowledge that some form of negative affective state (e.g., frustration, anger, humiliation) influences the person's reaction to the situation, socio-cognitive models propose that it generates aggression only to the extent that it influences cognitions, whereas psychodynamic place it at the very origin of aggressive impulses.

Similarly, in socio-cognitive models, motivations are fueled by internal reactions to external events, whereas Freud and Klein believed that they were relatively independent of from the social situation, and because Kohut proposed that the self was indistinguishable from the provoking object (in the environment), the internal/external divide here is also less significant.

Lastly, socio-cognitive models prioritize effortful, controlled processes that act to reduce negative affective states, redirect attention away from provoking stimuli, or suppress aggressive impulses. When overridden or defective, the combination of increase negative affect and a loss of control over cognitions increases the likelihood of an aggressive behavioral response. In contrast, while there is no self-initiated implementation of protective process in psychodynamic models (since the conflict takes place below conscious awareness), DMs are proposed to have different functions. Regarding the subsequent effects they have on aggressive impulses, depending on type, they can either increase, reduce, or have no effect at all.

Despite their differences, there are elements in each of these models that enrich our understanding of factors that underlie aggressive behavior. Importantly, psychodynamic models are personality models that were not developed to explain the problem of aggression *per se*, and socio-cognitive models of aggression [underpinned by Bandura's (1978) social learning theory] purposed to explain how socialization experiences contribute to patterns of thoughts and behaviors. As such, a possible benefit of integrating concepts of regulatory processes from both perspectives is that it opens new avenues for investigating aggression research using a more holistic approach.

Using an empirical approach in part 2, we examined associations between defense styles and prepotent response inhibition on their association with reactive aggression and the HIA. Finally, we also explored associations between the defense styles and response inhibition, as well as between individual DMs with aggression, HIA, and response inhibition. Results showed that reactive aggression and HIA were not significantly correlated with response inhibition but were significantly positively and negatively correlated with image distorting defense style and adaptive defense style, respectively. Moreover, certain individual DMs were significantly correlated with the inhibition index. Specifically, results suggested that response inhibition was correlated with a defense that has a similar function (stopping a direct aggressive response in passive aggression) rather than a mature defense (anticipation).

With this theoretical synthesis as well as the empirical findings in view, we propose an integrative framework that conceptualizes all the proposed psychological processes that influence aggression along a continuum of time, according to whether they are implemented before or after the aggressive act. To begin, several unconscious and/or automatic regulatory processes could occur before the aggressive act and result in an increase of negative affect (e.g., frustration, anger, humiliation). They would include image distorting DMs such as splitting, projective identification, and projection, as well as automatic cognitive processes such as aggressive priming, angry rumination, and HIA. When deployed, any one of these processes would increase the likelihood of an aggressive behavioral response. In parallel, processes resulting in a reduction of negative affect would comprise DMs such as repression, reaction formation, and sublimation, as well as cognitive inhibitory processes related to attention (e.g., ignoring salient/aversive features of the situation), emotion regulation (e.g., “calming down” by exerting control over negative feelings), resistance to proactive interference, and prepotent response inhibition. In the case that the person is consciously aware of the source of their aggressive impulse, then implementing these latter resources might also provide an opportunity for alternative interpretations of the situation (e.g., cognitive reappraisals). Lastly, in the case that the aggressive impulse was acted upon, DMs such as rationalization, suppression, and undoing, may help deescalate the situation by reducing negative affect, which may, in turn, reduce the risk of subsequent aggressive acts.

Although these hypotheses need to be verified with further experimental studies, they point to the prospect that converging multiple levels of analysis could further our efforts to unravel complex processes. Indeed, Westen (1991) made interesting propositions for integrating defense processes into socio-cognitive models of personality. According to him, contradictory representations of self and others could be associatively connected in long term-memory and yet blocked from consciousness because of their affective consequences, leading to the presence of activated but inaccessible nodes in an associative network. Similar theoretical integrations could be applied to socio-cognitive models of aggression.

As a result of having integrated knowledge from socio-cognitive and psychodynamic theories and research, this study provides insights and novel information. However, it also has limitations. First, the theoretical study was based on a small selection of socio-cognitive and psychodynamic models and was not intended to be representative of all theories of aggression within these fields. Regarding the empirical study, the first limitation relates to our reliance on a self-report questionnaire measure of DMs. Certain participants may have lacked insight regarding their defensive behaviors or had distorted or biased views of how they relate (or behave toward) others. Also, because of the correlational design of this study, we cannot make causal inferences about the relationships observed between DMs and aggression, HIA and response inhibition. For this to be possible, further development of experimental conditions that capture the effects of the DMs is necessary. The other limitation concerns our small sample, which may have prevented sufficient statistical power for verifying certain hypotheses.

## Conclusion

The present article has highlighted the importance of integrating socio-cognitive and psychodynamic models to account for the complexity underlying psychological processes that increase or decrease the likelihood of aggressive behavior. Notably, the theoretical part of the article identified two types of conflicts that play a critical role in whether processes are implemented. One is set at the conscious level and facilitates the recruitment of effortful, intentional control processes that promote conflict resolution by interrupting automatic thoughts and attenuating aggressive impulses. The other is set at the unconscious level and gives rise to defensive processes aiming to protect the self and resolve the conflict by either increasing or reducing the aggression. To integrate the processes taking place at these two levels of analysis, we proposed conceptualizing them along a continuum of time, according to whether they are implemented before or after the aggressive act. The empirical study included a sample of non-clinical participants and demonstrated that reactive aggression and the HIA were not significantly correlated with performance on a response inhibitory task but were significantly correlated with image distorting and adaptive defense style. Our results are consistent with previous research demonstrating significant relationships between aggression and defense styles among clinical samples and extend their findings to a non-clinical sample. Taken together with our integrative analysis, they provide insights for innovative studies exploring relationships between DMs and various inhibitory processes. Finally, our findings also suggest that, in addition to cognitive interventions for HABs, aggression prevention programs geared at young adults could benefit from interventions specifically targeted at increasing the use of mature DMs and decreasing the use of immature DMs.

## Data Availability Statement

The raw data supporting the conclusions of this article will be made available by the authors, without undue reservation.

## Ethics Statement

The studies involving human participants were reviewed and approved by the Comité d'Éthique de la Recherche en Psychologie et en Éducation (CEREP) (No. 2014-15-126-D). The patients/participants provided their written informed consent to participate in this study.

## Author Contributions

JG conceptualized the study and provided the data. JG, JQ, and PM analyzed and interpreted the data and contributed to the writing of the manuscript. All authors have approved the submitted version of this manuscript.

## Funding

This study was supported by a research grant to JG from the Social Sciences and Humanities Research Council (SSHRC) (No. 430-2014-00729).

## Conflict of Interest

The authors declare that the research was conducted in the absence of any commercial or financial relationships that could be construed as a potential conflict of interest.

## Publisher's Note

All claims expressed in this article are solely those of the authors and do not necessarily represent those of their affiliated organizations, or those of the publisher, the editors and the reviewers. Any product that may be evaluated in this article, or claim that may be made by its manufacturer, is not guaranteed or endorsed by the publisher.

## References

[B1] Acharya J. N. Hani A. Cheek J. Thirumala P. Tsuchida T. N. (2016). American clinical neurophysiology society guideline 2: guidelines for standard electrode position nomenclature. J. Clin. Neurophysiol. 33, 308–311. 10.1097/WNP.0000000000000316 27482794

[B2] American Psychiatric Association. (1994). Diagnostic and Statistical Manual of Mental Disorders. Washington, DC: Author.

[B3] American Psychiatric Association. (2013). Diagnostic and Statistical Manual of Mental Disorders: DSM-5. Washington, DC: American Psychiatric Association.

[B4] Anderson C. A. Bushman B. J. (2002). Human aggression. Ann. Rev. Psychol. 53, 27–51. 10.1146/annurev.psych.53.100901.135231 11752478

[B5] Anderson C. A. Carnagey N. L. (2004). Violent evil and the general aggression model. Soc. Psychol. Good Evil 4, 168–192.

[B6] Andrews G. Singh M. Bond M. (1993). The defense style questionnaire. J. Nerv. Ment. Dis. 181, 246–254. 10.1097/00005053-199304000-00006 8473876

[B7] Arsenio W. F. Adams E. Gold J. (2009). Social information processing, moral reasoning, and emotion attributions: relations with adolescents' reactive and proactive aggression. Child Develop. 80, 1739–1755. 10.1111/j.1467-8624.2009.01365.x 19930349

[B8] Axmacher N. Kessler H. Waldhauser G. T. (2014). Editorial on psychoanalytical neuroscience: exploring psychoanalytic concepts with neuroscientific methods. Front. Hum. Neurosci. 8:674. 10.3389/fnhum.2014.00674 25221501 PMC4147297

[B9] Bandura A. (1978). Social learning theory of aggression. J. Commun. 28, 12–29. 10.1111/j.1460-2466.1978.tb01621.x 690254

[B10] Barratt E. Stanford M. S. Kent T. A. Felthous A. (1997). Neuropsychological and cognitive psychophysiological subsrates of impulsive aggression. Biol. Psychiatr. 41, 1045–1061. 10.1016/S0006-3223(96)00175-8 9129785

[B11] Bartholow B. D. Fabiani M. Gratton G. Bettencourt B. A. (2001). A psychophysiological examination of cognitive processing of and affective responses to social expectancy violations. Psychol. Sci. 12, 197–204. 10.1111/1467-9280.00336 11437301

[B12] Bazan A. (2012). From sensorimotor inhibition to freudian repression: insights from psychosis applied to neurosis. Front. Psychol. 3:452. 10.3389/fpsyg.2012.00452 23162501 PMC3498871

[B13] Berkowitz L. (1993). Aggression: Its Causes, Consequences, and Control. New York, NY: McGraw-Hill.

[B14] Bond M. P. (1992). An empircal study of defensive styles: the defense style questionnaire, in Ego Mechanisms of Defense: A Guide for Clinicians and Researchers, ed Vaillant G. E. (Washington, DC: American Psychiatric Press), 127–158.

[B15] Bond M. P. Gardner S. T. Christian J. Sigal J. J. (1983). Empirical study of self-rated defenses styles. Archiv. Gene. Psychiatr. 40, 333–338. 10.1001/archpsyc.1983.01790030103013 6830412

[B16] Botvinick M. M. Braver T. S. Barch D. M. Carter C. S. Cohen J. D. (2001). Conflict monitoring and cognitive control. Psychol. Rev. 108, 624–652. 10.1037/0033-295X.108.3.624 11488380

[B17] Carlson M. Marcus-Newhall A. Miller N. (1990). Effects of situational aggression cues: a quantitative review. J. Personal. Soc. Psychol. 58:622. 10.1037/0022-3514.58.4.622 14570078

[B18] Cervone D. Shoda Y. (1999). The Coherence of Personality: Social-Cognitive Bases of Consistency, Variability, and Organization. New York, NY: Guilford Press.

[B19] Choe D. E. Lane J. D. Grabell A. S. Olson S. L. (2013). Developmental precursors of young school-age children's hostile attribution bias. Dev. Psychol. 49, 2245–2256. 10.1037/a0032293 23527492

[B20] Collins A. M. Loftus E. F. (1975). A spreading activation theory of semantic processing. Psychol. Rev. 82, 407–428. 10.1037/0033-295X.82.6.407

[B21] Cooper S. H. (1998). Changing notions of defense within psychoanalytic theory. J. Personal. 66, 947–964. 10.1111/1467-6494.0003816240612

[B22] Corruble E. Bronnec M. Falissard B. Hardy P. (2004). Defense styles in depressed suicide attempters. Psychiatr. Clinic. Neurosci. 58, 285–288. 10.1111/j.1440-1819.2004.01233.x 15149295

[B23] Cramer P. (1991). The Development of Defense Mechanisms: Theory, Research and Assessment. New York, NY: Springer Verlag.

[B24] Cramer P. Kelly F. D. (2004). Defense mechanisms in adolescent conduct disorder and adjustment reaction. J. Nerv. Mental Dis. 192:139. 10.1097/01.nmd.0000110285.53930.44 14770058

[B25] Crescioni A. W. Baumeister R. F. (2009). Alone and aggressive: Social exclusion impairs self-control and empathy and increases hostile cognition and aggression, in Bullying, Rejection, and Peer Victimization: A Social Cognitive Neuroscience Perspective, 251–277.

[B26] Crick N. R. Dodge K. A. (1994). A review and reformulation of social-information processing mechanisms in children's development. Psychol. Bull. 115, 74–101. 10.1037/0033-2909.115.1.74

[B27] Crick N. R. Dodge K. A. (1996). Social information-processing mechanisms in reactive and proactive aggression. Child Dev. 67, 993–1002. 10.2307/1131875 8706540

[B28] Delorme A. Makeig S. (2004). EEGLAB: an open source toolbox for analysis of single-trial EEG dynamics including independent component analysis. J. Neurosci. Methods 134, 9–21. 10.1016/j.jneumeth.2003.10.009 15102499

[B29] Denny K. G. Siemer M. (2012). Trait aggression is related to anger-modulated deficits in response inhibition. J. Res. Personal. 46, 450–454. 10.1016/j.jrp.2012.04.00125985979

[B30] di Giannantonio M. Northoff G. Salone A. (2020). Editorial: the interface between psychoanalysis and neuroscience: the state of the art. Font. Hum. Neurosci. 14, 1–2. 10.3389/fnhum.2020.00199 32581745 PMC7283447

[B31] Diamond A. (2013). Executive functions. Annu. Rev. Psychol. 64, 135–168. 10.1146/annurev-psych-113011-143750 23020641 PMC4084861

[B32] Dodge K. A. (1989). Coordinating responses to aversive stimuli: Introduction to a special section on the development of emotion regulation. Development. Psychol. 25:339. 10.1037/0012-1649.25.3.339

[B33] Dodge K. A. Coie J. D. (1987). Social-information-processing factors in reactive and proactive aggression in children's peer groups. J. Personal. Soc. Psychol. 53, 1146–1158. 10.1037/0022-3514.53.6.1146 3694454

[B34] Drisdelle B. L. Aubin S. Jolicoeur P. (2017). Dealing with ocular artifacts on lateralized ERPs in studies of visual-spatial attention and memory: ICA correction vs. epoch rejection. Psychophysiology 54, 83–99. 10.1111/psyp.12675 28000252

[B35] Eisenberg N. Zhou Q. Spinrad T. L. Valiente C. Fabes R. A. Liew J. (2005). Relations among positive parenting, children's effortful control, and externalizing problems: a three-wave longitudinal study. Child Dev. 76, 1055–1071. 10.1111/j.1467-8624.2005.00897.x 16150002 PMC1351058

[B36] Erdelyi M. H. (2006). The unified theory of repression. Behav. Brain Sci. 29, 499–511. 10.1017/S0140525X06009113 17156548

[B37] Fontaine R. G. Dodge K. A. (2006). Real-time decision making and aggressive behavior in youth: a heuristic model of response evaluation and decision (RED). Aggress. Behav. 32, 604–624. 10.1002/ab.20150 20802851 PMC2928648

[B38] Freud S. (1895). Project for a scientific psychology, in Standard Edition, 283–411.

[B39] Freud S. (1898). Sexuality in the Aetiology of the Neuroses, in Standard Edition, 259–285.

[B40] Freud S. (1905). Three essays on the theory of sexuality, in Standard Edition, 173–244.

[B41] Freud S. (1912). Recommendations to physicians practising psychoanalysis, in Standard Edition, 109–120.

[B42] Freud S. (1915a). Instincts and their vicissitudes, in Standard Edition, 103–196.

[B43] Freud S. (1915b). Repression, in Standard Edition, 103–196.

[B44] Freud S. (1920). Beyond the pleasure principle, in Standard Edition, 1–65.

[B45] Freud S. (1923). The Ego and the Id, in Standard Edition, 3–69.

[B46] Friedman N. P. Miyake A. (2004). The relations among inhibition and interference control functions: a latent-variable analysis. J. Exp. Psychol. Gen. 133, 101–135. 10.1037/0096-3445.133.1.101 14979754

[B47] Gagnon J. Aubin M. Carrier Emond F. Derguy S. Bessette M. Jolicoeur P. (2016). Neural mechanisms underlying attribution of hostile intention in non-aggressive individuals: an ERP study. Int. J. Psychophysiol. 110, 153–162. 10.1016/j.ijpsycho.2016.08.007 27543324

[B48] Gagnon J. Aubin M. Emond F. C. Derguy S. Brochu A. F. Bessette M.. (2017). An ERP study on hostile attribution bias in aggressive and non-aggressive individuals. Aggress. Behav. 43, 217–229. 10.1002/ab.21676 27629652

[B49] Gagnon J. Jolicœur P. (2014). Mécanismes cognitifs et neurophysiologiques associés au trait “Urgence” de la personnalité: intégration théorique et empirique, in Insight Development: Social Sciences and Humanities Research Council of Canada.

[B50] Gagnon J. Quansah J. Kim W. (2019). When aggression is out of control: from one-person to two-person neuropsychology. Inhibit. Control Train. Multidisciplin. Approach. 19:89803. 10.5772/intechopen.89803

[B51] Gagnon J. Rochat L. (2017). Relationships between hostile attribution bias, negative urgency, and reactive aggression. J. Individ. Differen. 38:211. 10.1027/1614-0001/a00023825309304

[B52] Gilbert F. Daffern M. Anderson C. A. (2017). The general aggression model and its application to violent offender assessment and treatment, in The wiley handbook of violence and aggression, 1-13.

[B53] Gleser G. C. Ihilevich D. (1969). An objective instrument for measuring defense mechanisms. J. Consult. Clinic. Psychol. 33, 51–60. 10.1037/h0027381 5776301

[B54] Godleski S. A. Ostrov J. M. Houston R. J. Schlienz N. J. (2010). Hostile attribution biases for relationally provocative situations and event-related potentials. Int J Psychophysiol. 76, 25–33. 10.1016/j.ijpsycho.2010.01.010 20117151

[B55] Goeleven E. De Raedt R. Leyman L. Verschuere B. (2008). The Karolinska directed emotional faces: a validation study. Cogn. Emot. 22, 1094–1118. 10.1080/0269993070162658229312053

[B56] Hann N. (1977). Coping and Defending: Processes Of Self-Environment Organization. New York, NY: Academy Press.

[B57] Hawkins K. A. Cougle J. R. (2013). Effects of interpretation training on hostile attribution bias and reactivity to interpersonal insult. Behav. Ther. 44, 479–488. 10.1016/j.beth.2013.04.005 23768674

[B58] Hovanesian S. Cervellione K. L. (2009). Defense mechanisms and suicide risk in major depression. Archiv. Suicide Res. 13, 74–86. 10.1080/13811110802572171 19123111

[B59] Hsieh I. J. Chen Y. Y. (2017). Determinants of aggressive behavior: Interactive effects of emotional regulation and inhibitory control. PLoS ONE 12:e0175651. 10.1371/journal.pone.0175651 28399150 PMC5388499

[B60] Ireland J. L. Archer J. (2002). The perceived consequences of responding to bullying with aggression: A study of male and female adult prisoners. Aggress. Behav. Offic. J. Int. Soc. Res. Aggress. 28, 257–272. 10.1002/ab.8000125855820

[B61] John O. P. Gross J. J. (2004). Healthy and unhealthy emotion regulation: personality processes, individual differences, and life span development. J. Pers. 72, 1301–1333. 10.1111/j.1467-6494.2004.00298.x 15509284

[B62] Joseph B. (1983). On understanding and not understanding: some technical issues. Int. J. Psychoanal. 64, 291–298. 6618778

[B63] Kalanthroff E. Cohen N. Henik A. (2013). Stop feeling: inhibition of emotional interference following stop-signal trials. Front. Hum. Neurosci. 7:78. 10.3389/fnhum.2013.00078 23503817 PMC3596782

[B64] Kalisch R. Wiech K. Herrmann K. Dolan R. J. (2006). Neural correlates of self-distraction from anxiety and a process model of cognitive emotion regulation. J. Cogn. Neurosci. 18, 1266–1276. 10.1162/jocn.2006.18.8.1266 16859413 PMC2638061

[B65] Klein M. (1946). Notes on some schizoid mechanisms. Int. J. Psychoanal. 27, 99–110. 20261821

[B66] Klein M. (1975a). Envy and Gratitude an Other Works 1946–1963 (The Writings of Melanie Klein, Vol. 3). London: Hogarth Press.

[B67] Klein M. (1975b). Love, Guilt and Reparation and Other Works 1921–1945 (The Writings of Melanie Klein, Vol. 1). London: Hogarth Press.

[B68] Klein M. (1975c). The Psycho-Analysis of Children (1932) (The Writings of Melanie Klein, Vol. 2). London: Hogarth Press.

[B69] Koenigsberg H. W. Harvey P. D. Mitropoulou V. New A. S. Goodman M. Silverman J.. (2001). Are the interpersonal and identity disturbances in the borderline personality disorder criteria linked to the traits of affective instability and impulsivity? J. Personal. Disord. 15, 358–370. 10.1521/pedi.15.4.358.19181 11556702

[B70] Kohut H. (1966). Forms and transformations of narcissism. J. Am. Psychoanal. Assoc. 14, 243–272. 10.1177/000306516601400201 5941052

[B71] Kohut H. (1971). The Analysis of the Self . New York, NY: International Universities Press.

[B72] Kohut H. (1972). Thoughts on narcissism and narcissistic rage. Psychoanal. Study Child 27, 360–400. 10.1080/00797308.1972.118227213984534

[B73] Kohut H. (1977). The Restoration of the Self . New York, NY: International Universities Press.

[B74] Kutas M. Federmeier K. D. (2011). Thirty years and counting: finding meaning in the N400 component of the event-related brain potential (ERP). Ann. Rev. Psychol. 62, 621–647. 10.1146/annurev.psych.093008.131123 20809790 PMC4052444

[B75] Laplanche J. Pontalis J. B. (2018). The Language of Psycho-Analysis (6th ed.). New York, NY: Routledge.

[B76] Lee Y. J. Keum M. S. Kim H. G. Cheon E. J. Cho Y. C. Koo B. H. (2020). Defense mechanisms and psychological characteristics according to suicide attempts in patients with borderline personality disorder. Psychiatric Investigat. 17, 840–849. 10.30773/pi.2020.0102 32791818 PMC7449843

[B77] Leuthold H. Filik R. Murphy K. Mackenzie I. G. (2012). The on-line processing of socio-emotional information in prototypical scenarios: inferences from brain potentials. Soc. Cogn. Affect Neurosci. 7, 457–466. 10.1093/scan/nsr029 21609967 PMC3324574

[B78] Lingiardi V. McWilliams N. (2017). Psychodynamic Diagnostic Manual - second edition (PDM-2). New York, NY: Guilford. 10.1002/wps.20233 PMC4471982 26043343

[B79] Liu Y. Zhan X. Li W. Han H. Wang H. Hou J.. (2014). The trait anger affects conflict inhibition: a Go/Nogo ERP study. Front. Hum. Neurosci. 8:1076. 10.3389/fnhum.2014.01076 25653610 PMC4301017

[B80] Lochman J. E. Wells K. C. (2002). Contextual social-cognitive mediators and child outcome: a test of the theoretical model in the Coping Power program. Dev. Psychopathol. 14, 945–967. 10.1017/S0954579402004157 12549711

[B81] Lopez-Calderon J. Luck S. J. (2014). ERPLAB: an open-source toolbox for the analysis of event-related potentials. Front. Hum. Neurosci. 8:213. 10.3389/fnhum.2014.00213 24782741 PMC3995046

[B82] Luck S. J. (2014). An Introduction to the Event-related Potential Technique. New York, NY: MIT press.

[B83] Mischel W. Shoda Y. (1995). A cognitive-affective system theory of personality: reconceptualizing situations, dispositions, dynamics, and invariance in personality structure. Psychol. Rev. 102:246. 10.1037/0033-295X.102.2.246 7740090

[B84] Oosterlaan J. Sergeant J. A. (1996). Inhibition in ADHD, aggressive, and anxious children: a biologically based model of child psychopathology. J. Abnorm Child Psychol. 24, 19–36. 10.1007/BF01448371 8833026

[B85] Osgood J. M. Kase S. E. Zaroukian E. G. Quartana P. J. (2021). Online intervention reduces hostile attribution bias, anger, aggressive driving, and cyber-aggression, results of two randomized trials. Cogn. Therap. Res. 45, 310–321. 10.1007/s10608-020-10147-8

[B86] Pawliczek C. M. Derntl B. Kellermann T. Kohn N. Gur R. C. Habel U. (2013). Inhibitory control and trait aggression: neural and behavioral insights using the emotional stop signal task. NeuroImage 79, 264–274. 10.1016/j.neuroimage.2013.04.104 23660028

[B87] Perry C. (1990). Defense Mechanisms Rating Scales. Cambridge, MA: The Cambridge Hospital.

[B88] Petraglia J. Thygesen K. L. Lecours S. Drapeau M. (2009). Gender differences in self-reported defense mechanisms: a study using the new Defense Style Questionnaire-60. Am. J. Psychother. 63, 87–99. 10.1176/appi.psychotherapy.2009.63.1.87 19425336

[B89] Posner M. I. Rothbart M. K. (2000). Developing mechanisms of self-regulation. Develop. Psychopathol. 12, 427–441. 10.1017/S0954579400003096 11014746

[B90] Puhalla A. A. McCloskey M. S. Brickman L. J. Fauber R. Coccaro E. F. (2016). Defense styles in intermittent explosive disorder. Psychiatry Res. 238, 137–142. 10.1016/j.psychres.2016.02.019 27086223

[B91] Raine A. Dodge K. Loeber R. Gatzke-Kopp L. Lynam D. Reynolds C.. (2006). The reactive-proactive aggression questionnaire: differential correlates of reactive and proactive aggression in adolescent boys. Aggress. Behav. 32, 159–171. 10.1002/ab.20115 20798781 PMC2927832

[B92] Rebetez M. M. Rochat L. Billieux J. Gay P. Van der Linden M. (2015). Do emotional stimuli interfere with two distinct components of inhibition? Cogn. Emot. 29, 559–567. 10.1080/02699931.2014.922054 24885111

[B93] Rudolph U. Roesch S. C. Greitemeyer T. Weiner B. (2004). A meta-analytic review of help giving and aggression from an attributional perspective: contributions to a general theory of motivation. Cognition and Emotion. 18, 815–848. 10.1080/02699930341000248

[B94] Set Z. Ergin Ö. (2020). The investigation of the mediator effect of sexism and defense style in the relationship between homophobia and aggression. Archiv. Neuropsychiatr. 57, 113–119. 10.29399/npa.24743 32550776 PMC7285642

[B95] Shevrin H. Bond J. A. Brakel L. A. Hertel R. K. Williams W. J. (1996). Conscious and Unconscious Processes: Psychodynamic, Cognitive, and Neurophysiological Convergences. New York, NY: Guilford Press.

[B96] Shevrin H. Snodgrass J. M. Brakel L. A. W. Kushwaha R. Kalaida N. L. Bazan A. (2013). Subliminal unconscious conflict alpha power inhibits supraliminal conscious symptom experience. Front. Hum. Neurosci. 7, 1–12. 10.3389/fnhum.2013.00544 24046743 PMC3763585

[B97] Shevrin H. Williams W. J. Marshall R. E. Hertel R. K. Bond J. A. Brakel L. A. (1992). Event-related potential indicators of the dynamic unconscious. Conscious. Cogn. 1, 340–366. 10.1016/1053-8100(92)90068-L

[B98] Stanford M. S. Houston R. J. Mathias C. W. Villemarette-Pittman N. R. Helfritz L. E. Conklin S. M. (2003). Characterizing aggressive behavior. Assessment 10, 183–190. 10.1177/1073191103010002009 12801190

[B99] Stewart J. L. Silton R. L. Sass S. M. Fisher J. E. Edgar J. C. Heller W.. (2010). Attentional bias to negative emotion as a function of approach and withdrawal anger styles: an ERP investigation. Int. J. Psychophysiol. 76, 9–18. 10.1016/j.ijpsycho.2010.01.008 20109502 PMC2867457

[B100] Sun L. Li J. Niu G. Zhang L. Chang H. (2020). Reactive aggression affects response inhibition to angry expressions in adolescents: an event-related potential study using the emotional go/no-go paradigm. Front Psychol. 11, 558461. 10.3389/fpsyg.2020.558461 33101129 PMC7556161

[B101] Thygesen K. L. Drapeau M. Trijsburg R. W. Lecours S. de Roten Y. (2008). Assessing defense styles: factor structure and psychometric properties of the new defense style questionnaire 60 (DSQ-60). Int. J. Psychol. Psychol. Therap. 8, 171–181.

[B102] Vaillant G. E. (1976). Natural history of male psychological health: the relation of choice of ego mechanisms of defense to adult adjustment. Archiv. General Psychiatry 33, 535–545. 10.1001/archpsyc.1976.01770050003001 1267569

[B103] Vaillant G. E. (1987). A developmental view of old and new perspectives of psychology disorders. J. Personal. Disorders 1, 146–156. 10.1521/pedi.1987.1.2.146

[B104] Vaillant G. E. (1992). Ego Mechanisms of Defense. Washington, DC: American Psychiatric Press.

[B105] van Adrichem D. S. Huijbregts S. C. J. van der Heijden K. B. van Goozen S. H. M. Swaab H. (2020). The role of inhibitory control, attention and vocabulary in physical aggression trajectories from infancy to toddlerhood. Front Psychol 11:1079. 10.3389/fpsyg.2020.01079 32528388 PMC7264375

[B106] Verbruggen F. De Houwer J. (2007). Do emotional stimuli interfere with response inhibition? evidence from the stop signal paradigm. Cogn. Emot. 21, 391–403. 10.1080/02699930600625081

[B107] Verbruggen F. Logan G. D. (2008). Response inhibition in the stop-signal paradigm. Trends Cogn. Sci. 12, 418–424. 10.1016/j.tics.2008.07.005 18799345 PMC2709177

[B108] Verbruggen F. Logan G. D. Stevens M. A. (2008). STOP-IT: Windows executable software for the stop-signal paradigm. Behav. Res. Methods 40, 479–483. 10.3758/BRM.40.2.479 18522058

[B109] Verona E. Bresin K. (2015). Aggression proneness: Transdiagnostic processes involving negative valence and cognitive systems. Int. J. Psychophysiol. 98, 321–329. 10.1016/j.ijpsycho.2015.03.008 25816797

[B110] Westen D. (1991). Social cognition and object relations. Psychol. Bull. 109:429. 10.1037/0033-2909.109.3.429

[B111] Whitman C. N. Gottdiener W. H. (2015). Implicit coping styles as a predictor of aggression. J. Aggress. Maltreat. Trauma 24, 809–824. 10.1080/10926771.2015.1062447

[B112] Wilkowski B. M. Robinson M. D. (2008). The cognitive basis of trait anger and reactive aggression: an integrative analysis. Pers. Soc. Psychol. Rev. 12, 3–21. 10.1177/1088868307309874 18453470

[B113] Wilkowski B. M. Robinson M. D. (2010). The anatomy of anger: an integrative cognitive model of trait anger and reactive aggression. J. Pers. 78, 9–38. 10.1111/j.1467-6494.2009.00607.x 20433611

[B114] Zelli A. Rowell Huesmann L. Cervone D. (1995). Social inference and individual differences in aggression: evidence for spontaneous judgments of hostility. Aggress. Behav. 21, 405–417. 10.1002/1098-2337(1995)21:6<405::AID-AB2480210602>3.0.CO;2-N

